# Cross-species analysis of FcγRIIa/b (CD32a/b) polymorphisms at position 131: structural and functional insights into the mechanism of IgG- mediated phagocytosis in human and macaque

**DOI:** 10.3389/fimmu.2025.1726068

**Published:** 2025-12-08

**Authors:** William D. Tolbert, Paula B. Nhan, Haleigh E. Conley, Xiaoxuan Ge, Monika Chandravanshi, Madeleine Lee, Julianna Veilleux, Marek Korzeniowski, Suneetha Gottumukkala, Margaret E. Ackerman, Justin Pollara, Marzena Pazgier

**Affiliations:** 1 Infectious Disease Division, Department of Medicine of the Uniformed Services University of the Health Sciences, Bethesda, MD, United States; 2 Henry M. Jackson Foundation for the Advancement of Military Medicine, Inc., Bethesda, MD, United States; 3 Department of Surgery, Duke University School of Medicine, Durham, NC, United States; 4 Thayer School of Engineering, Dartmouth College, Hanover, NH, United States

**Keywords:** human *Homo sapiens*, FcγRIIa His/Arg131, rhesus macaque *Macaca mulatta*, FcγRIIa His/Pro131, CD32, Fc-effector function, IgG1(Fc)-FcγRII complex structure, IgG2(Fc)-FcγRIIa complex structure

## Abstract

**Introduction:**

Antibodies play a critical role in immunity in part by mediating clearance of pathogens and infected cells by antibody-dependent cellular phagocytosis (ADCP) through engagement of Fc gamma receptors (FcγRs) on innate immune cells. Among these, FcγRIIa (CD32a) is a key activating receptor expressed on macrophages, dendritic cells, and other antigen-presenting cells. Its affinity for IgG and ability to mediate ADCP is influenced by allelic polymorphisms. In humans, a single amino acid polymorphism at position 131, where histidine (H) is substituted with arginine (R), leads to decreased IgG1 and IgG2 subclass binding affinity and, consequently, lower efficiency of phagocytic responses. Rhesus macaques (*Macaca mulatta*), which are widely used as nonhuman primate models, exhibit a similar polymorphism at position 131 of FcγRIIa, but with arginine replaced by proline (P). Here, we investigated structure-function relationships associated with the FcγRIIa polymorphism at position 131 in both species, specifically with respect to IgG1 and IgG2.

**Methods:**

We determined the structures of complexes formed by each variant with IgG1 Fc and those formed by the higher affinity variant with IgG2 Fc for both species by x-ray crystallography and linked these structures to affinity and activity using SPR and an ADCP assay. We also determined the structure of human inhibitory FcγRIIb (CD32b) in complex with IgG1 Fc by x-ray crystallography.

**Results:**

Through analysis of these structures, our studies reveal that FcγRIIa engagement is minimally influenced by Fc glycan composition, distinguishing it from FcγRIIIa whose affinity is strongly influenced by glycan-composition. Comparative structures of human and macaque FcγRIIa variants demonstrate species- and allele-specific differences in Fc binding, but our functional assays showed only minimal allele-specific effects in humans. In contrast, allele-specific effects in macaques were highly significant; the macaque P^131^ variant showing uniformly reduced IgG affinity.

**Conclusion:**

These insights highlight fundamental interspecies and allelic distinctions that are critical for interpreting FcγRIIa-mediated effector functions in macaque models and for optimizing translational antibody and vaccine design.

## 1 Introduction

In humans, *homo sapiens* (*Hs*), the four IgG subclasses of immunoglobulin G (IgG) account for approximately 75% of the total immunoglobulin in serum. IgG exists as four subclasses, IgG1, IgG2, IgG3, and IgG4, originally named in order of their relative abundance in circulation (IgG1-4) (1). These subclasses differ in their hinge region structure, flexibility, serum half-life, ability to activate complement, and affinity for Fc gamma receptors (FcγRs), which together influence their effector function profiles. There are five activating FcγRs in humans, FcγRI (CD64), FcγRIIa (CD32a), FcγRIIc (CD32c) – present in only a fraction of individuals due to the presence of a stop codon in most individuals, FcγRIIIa (CD16a) and FcγRIIIb (CD16b), and one inhibitory receptor, FcγRIIb (CD32b) (2). Rhesus macaques, *Macaca mulatta* (*Mm*), also have four IgG subclasses, however, as each is most similar to human IgG1, they are not perfect equivalents to human IgG1-4, either in sequence identity or functional properties. For example, macaque IgG3 lacks the hallmark feature of human IgG3, a long hinge region that contributes to its unique flexibility and effector function profile (3, 4). Rhesus macaques also differ from humans in their FcγR repertoire; they lack two of the activating receptors, FcγRIIc and FcγRIIIb, due to a gene duplication event that occurred only in higher primates (5). Activation of Fcγ receptors leads to downstream host cell functions such as antibody-dependent cellular phagocytosis (ADCP) and antibody-dependent cellular cytotoxicity (ADCC) collectively known as Fc-dependent effector functions – the Fc portion of the IgG binds the receptor (6, 7). The type and distribution of Fcγ receptors on the immune cell surface determines to a large extent how those cells will respond to IgG stimulus.

FcγRIIa is expressed on macrophages, dendritic cells and other antigen presenting cells and is largely associated with ADCP. FcγRIIa is unique in that it has its own immunoreceptor tyrosine-based activation motif (ITAM) and does not rely on the γ-chain for signaling (5). Within the context of HIV-1, the HVTN 505 HIV-1 clinical trial showed a correlation between anti-Env serum IgG3, ADCP and *in vitro* FcγRIIa engagement and a reduced risk of HIV-1 infection and a correlation between FcγRIIa engagement and a decreased viral load setpoint in breakthrough vaccinees (8). Although HVTN 505, a DNA and Adenovirus 5 vaccine regimen, did not meet its overall efficacy endpoint criteria, it did suggest an important role for FcγRIIa in protection against HIV-1. FcγRIIa has also been linked to protection against capsulated bacterial infection, specifically with respect to carbohydrate antigens (9), and to the “vaccinal effect”, the long-lasting protective effect of therapeutic antibodies in cancer beyond their presence in serum which is thought to be due to FcγRIIa engagement on antigen presenting dendritic cells (10).

Humans have a polymorphism at position 131 in the extracellular domain of the receptor that can affect receptor affinity to IgG, especially with respect to the IgG2 subclass. FcγRIIa with arginine (R) at position 131 binds IgG2 poorly while FcγRIIa with histidine (H) at position 131 binds IgG2 better (11). Interestingly, macaques share a FcγRIIa polymorphism with human with respect to position 131 but not with respect to the amino acid identity, as in macaques histidine is replaced by proline (P) instead of arginine (12, 13). Macaque FcγRIIa with histidine at position 131 binds macaque IgG of all subclasses with higher affinity than FcγRIIa with proline at position 131. The impact of this polymorphism on function may be reflected in its population distribution. H^131^ and R^131^ alleles are generally equally prevalent, resulting in roughly half of the population being heterozygous and the other half homozygous for one of the two alleles (roughly 25% H^131^ and 25% R^131^). Two exceptions to this are historically Asian populations which are roughly 50% homozygous for the H^131^ allele and Amazonian Indian populations which are roughly 84% homozygous for the R^131^ allele (14). Conversely, the P^131^ prevalence in macaques is much lower with only approximately 2.5% of individuals having at least one copy of the P^131^ allele (13).

Given the central role of FcγRIIa in mediating ADCP and its relevance to protective immunity, we aimed to elucidate how the polymorphism at position 131 influences FcγRIIa affinity to IgG subclasses, the structural properties of species-matched Fc-receptor complexes, and function in both humans and rhesus macaques. For each species, we determined the crystal structures of both receptor variants in complex with IgG1 Fc, as well as the structures of the higher-affinity variants bound to IgG2 Fc. We also determined the structure of the complex of human FcγRIIb in complex with human IgG1 Fc. In parallel, we analyzed the binding affinities of human and macaque IgG subclasses to their corresponding FcγRIIa variants and assessed their ability to mediate ADCP using primary human monocytes homozygous for either the human H^131^ or R^131^ receptor variant. Our data reveal distinct differences in subclass binding and functional activity between the species, pointing to interspecies variation in FcγRIIa-IgG interactions that could influence the interpretation of preclinical vaccine and antibody efficacy studies conducted in rhesus macaques.

## 2 Materials and methods

### 2.1 Protein expression and purification

Human receptors were expressed in HEK 293 cells and purified on IgG sepharose (Cytiva). Macaque receptors were expressed in CHO cells and purified using nickel magnetic beads (GenScript). IgGs were expressed in Expi293 cells (ThermoFisher) by transfection of heavy and light chain plasmids and were purified from medium on protein A (Cytiva). Receptors and IgGs were further purified by gel filtration chromatography on a Superdex 200 increase 10/300 column (Cytiva) equilibrated in phosphate buffered saline (PBS) pH 7.4.

### 2.2 Antibody-dependent cellular phagocytosis assay

Fluorescent HIV virions were generated by transfection of 293T cells (ATCC #CRL-3216) with plasmid encoding HIV NL4–3 JRFL iGFP (NIH HIV Reagent Program, BEI Resources) using PolyFect transfection reagent (Qiagen). A media change was performed 6 hours post-transfection. The supernatant was harvested 48 hours later and subsequently filtered through a 0.45 µm PVDF filter (Millipore Sigma), aliquoted, and frozen at -80°C in 20% fetal bovine serum (FBS) supplemented cell culture medium. Immune complexes containing fluorescently-labeled HIV NL4–3 JRFL iGFP were generated by plating 10 µL of virus with 10 µL of monoclonal antibodies at a final concentration of 10, 1, or 0.1 µg/mL in 96-well round bottom plates for 2 hours at 37°C (15). Human peripheral venous blood was collected by leukapheresis from healthy consenting adult volunteers in accordance with the policies and regulations of the Duke Health Institutional Review Board. Monocytes were enriched from peripheral blood mononuclear cells (PBMCs), previously genotyped for FCGR2A (FcγRIIa) allelic polymorphisms at position 131, using negative selection with magnetic beads (Pan Monocyte kit, Miltenyi) according to the manufacturer’s instructions. Monocytes were treated with 10 µg/mL anti-CD4 (Biolegend, clone OKT4) at a cell concentration of 1x10^7^ cells/mL in RPMI-1640 + 1% FBS for 15 minutes at 4°C. Cells were then resuspended to a density of 5x10^5^ cells/mL in RPMI-1640 + 1% FBS, and 50,000 cells (100 µL) were added to immune complexes. Cells were then centrifuged for 1 hour at 1200 x g at 4°C and incubated for 1 hour at 37°C with 5% CO_2_. They were then washed in 1% FBS PBS and fixed with a 4% formaldehyde PBS solution. Data acquisition and data analysis were performed using a BD LSRFortessa flow cytometer and FlowJo Software. ADCP activity is presented as a phagocytosis score, calculated as the percentage of GFP positive cells x median fluorescence intensity (MFI) of GFP, normalized by the corresponding result for the no-antibody control.

### 2.3 Complex preparation for structural studies

IgG Fcs were generated by papain digest of IgGs (human IgG1 and IgG2) or expressed in Expi293 cells as Fc constructs (macaque IgG1 and IgG2). Briefly, papain digest consisted of incubation of IgG with immobilized papain (Thermo Fisher) for 3–4 hours at 37°C in phosphate buffer pH 7.0 supplemented with 3.5 mg/ml L-cysteine. Papain was then removed by centrifugation. Fcs were separated from Fab (in the case of IgG digest) or purified from medium (in the case of Fc expression) on protein A. Papain digest or medium was passed over a HiTrap protein A affinity column (Cytiva) equilibrated in PBS pH 7.4. The column was then washed with PBS pH 7.4. Fc/IgG was eluted with 0.1 M glycine pH 3.0. Undigested IgG (in the case if IgG digest) was separated from Fc by gel filtration using a Superdex 200 increase 10/300 column (Cytiva) equilibrated in PBS pH 7.4 (**Supplementary Figure S1**). Complexes were made with an excess of Fc, which was removed by purification by gel filtration using a Superdex 200 increase 10/300 column (Cytiva), and were then used to grow protein crystals for X-ray structure determination.

### 2.4 Crystallization and structure determination

The buffer of the complexes was exchanged from PBS to 25 mM Tris-HCl pH 7.2 and 100 mM ammonium acetate using Amicon centrifugal concentrators, and the protein was the concentrated to approximately 10 mg/ml in preparation for crystallization. Crystals were grown by the hanging drop method. Briefly, protein and crystallization condition were mixed at a ratio of 1:1 on a siliconized glass coverslip and then sealed over a well containing the crystallization condition (0.5 mL) in 24 well crystallization plates (Hampton Research). Commercial crystallization screens from Molecular Dimensions, ProPlex Eco and MacroSol Eco, were used to determine initial crystallization conditions producing crystals which were then optimized manually. Macaque IgG1Fc-FcγRIIa(H^131^) crystals were grown from 15% polyethylene glycol (PEG) and 0.1 M HEPES pH 7.0, macaque IgG2Fc-FcγRIIa(H^131^) crystals were grown from 12% PEG 8000, 0.1 M magnesium acetate, and 0.1 M MOPS pH 7.5, and macaque IgG1Fc-FcγRIIa(P^131^) crystals were grown from 10% PEG 5000 monomethyl ether (MME), 12% isopropanol, and 0.1 M MES pH 6.5. Human IgG1Fc-FcγRIIa(H^131^) and IgG2Fc-FcγRIIa(H^131^) crystals were grown from 25% PEG 2000 MME and 0.1 M HEPES pH 7.5 and human IgG1Fc-FcγRIIa(R^131^) crystals were grown from 10% PEG 5000 MME, 12% isopropanol, and 0.1 M MES pH 6.5. Human IgG1Fc-FcγRIIb crystals were grown from 8% PEG 6000, 100 mM Tris-HCl pH 8.0, and 150 mM sodium chloride. All crystals were cryoprotected with 20% 2-methyl-2,4-pentanediol (MPD) prepared in the respective crystallization condition and flash frozen in liquid nitrogen for data collection. Data were collected at the Stanford Synchrotron Radiation Lightsource (SSRL) beamlines 12–1 and 12-2.

Structures were solved by molecular replacement using the program PHASER from the CCP4 program suite (16, 17). The initial receptor model was from PDB ID 3RY6, and the initial Fc model was from PDB ID 3AVE or PDB ID 6D4E for the human or macaque complexes, respectively. Model building was done with COOT (18) and refinement was done with PHENIX (19). Model quality was monitored with Molprobity (20). Data collection and refinement statistics are provided in **Supplementary Table S2**.

### 2.5 Surface plasmon resonance

IgG subclass binding to FcγRIIa and FcγRIIb was evaluated by surface plasmon resonance. Human and macaque antibodies were covalent linked to a carboxymethyl dextran-functionalized biosensor (CMD200M, Xantec Bioanalytics) using a continuous flow microspotter (Carterra) capable of producing 96 discrete regions of interest. The microfluidic pathways were primed with 10 mM sodium acetate (pH 5.0) and activated for 5 minutes with 100 µL of 10.4 mM 1-ethyl-3-(3-dimethylaminopropyl)carbodiimide (EDC) and 1.4 mM N-hydroxysulfosuccinimide (sulfo-NHS) (ThermoFisher) prepared in 10 mM MES (pH 5.0). Each antibody was prepared in 10 mM sodium acetate (pH 5.0) at concentrations of 100 nM, 200 nM, 300 nM, and 400 nM as three or four technical replicates and passed over the activated regions for 7 minutes, followed by 5 minutes of wash with sodium acetate. The sensor chip was loaded on a PBS + 0.05% Tween 20-primed imaging-based SPR (MX96, IBIS Technologies) and quenched with a 150 µL injection of 1 M ethanolamine (pH 8.5). Each FcγRII receptor was formulated at 30 µM in PBS + 0.05% Tween 20 and serially diluted over an 8-point 3-fold dilution series. The association time between printed IgG and each FcγR was set for 200 s, and disassociation time for 300 s. Between each FcγR concentration, the chip was regenerated using 10 mM glycine, pH 2.7, for 30 s and then primed with PBS + 0.05% Tween 20 for 30 s. The instrument was maintained at 25 °C throughout the experiment. Initial processing of the SPR data was performed using SprintX (IBIS Technologies). The signal from each region of interest on the sensor was referenced using the nearest unconjugated inter spot to account for bulk shifts and nonspecific binding. The blank injection immediately preceding each series of receptors was subtracted from the signal of each of the injections within a receptor series. Affinity values were calculated in Scrubber 2 (BioLogic Software) using k_on_ and k_off_ values from the best kinetic fit to 1:1 binding stoichiometry.

## 3 Results

### 3.1 FcγRIIa residue 131 polymorphisms differentially impact IgG subclass binding in humans and rhesus macaques

**Figure 1:**
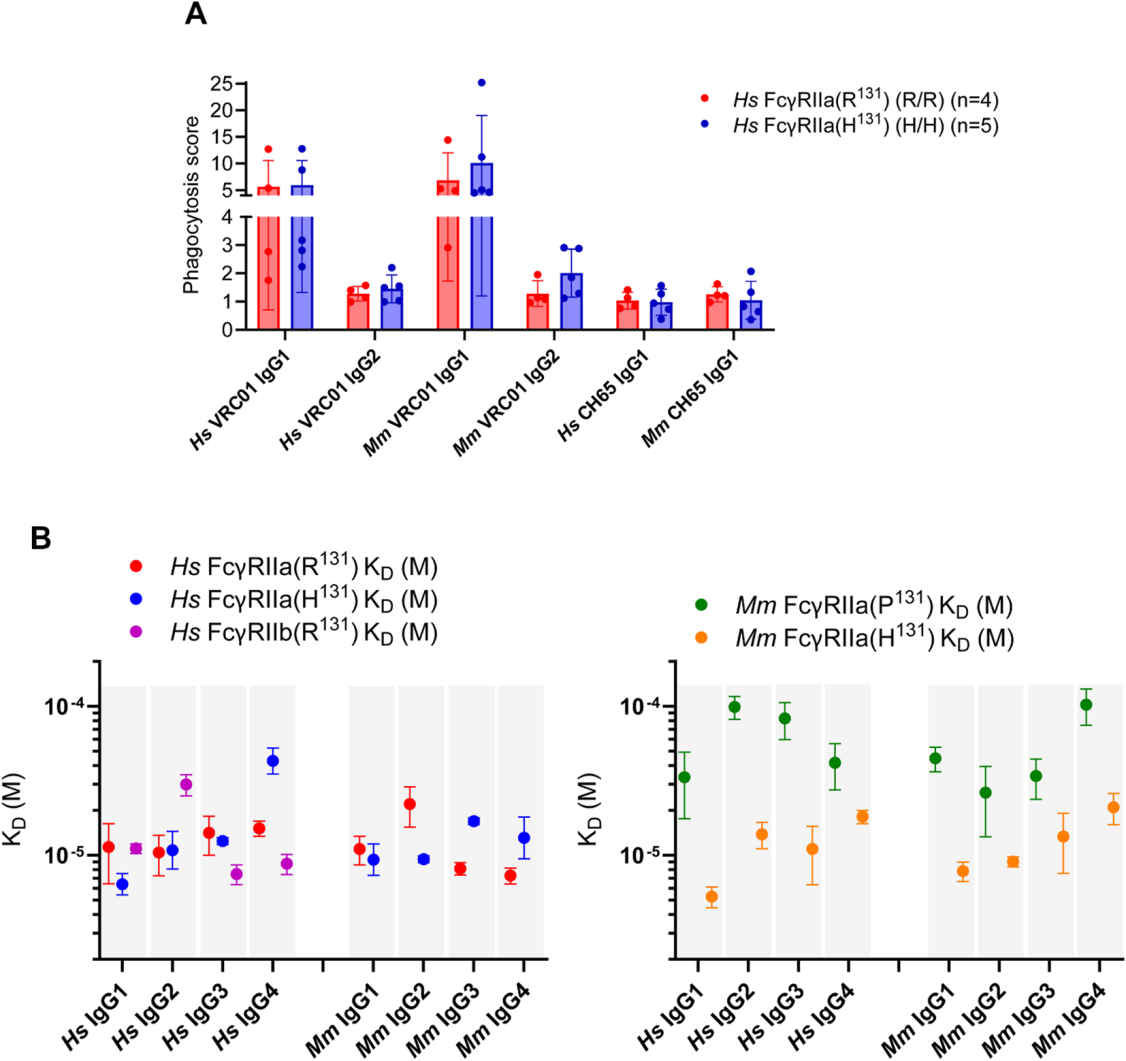
ADCP activity and K_D_ values. **(A)** ADCP activity measured as a phagocytosis score (mean plus or minus standard deviation) for human and macaque VRC01 IgG1 and IgG2 using human monocytes homozygous for the R^131^ (n=4) or H^131^ (n=5) FcγRIIa allele. The human influenza CH65 IgG1 is used as a control. **(B)** K_D_ values for human and macaque VRC01 IgG1, 2, 3, and 4 against human and macaque FcγRIIa variants, H^131^ and R^131^ for human and H^131^ and P^131^ for macaque, and the human FcγRIIb. K_D_ values are plotted as the mean ± the standard deviation from three or four technical replicates for each antibody-FcγR interaction. Binding affinities were assessed by SPR with IgG as the ligand and receptor as the analyte.

To examine the effect of the FcγRIIa polymorphism on function, we compared the ADCP activity of primary human monocytes isolated from four individuals homozygous for the R^131^ allelic variant of FcγRIIa, and five individuals homozygous for H^131^ variant. Immune complexes were formed with green fluorescent protein (GFP)-expressing HIV virions and the HIV-1 CD4 binding site specific mAb VRC01, recombinantly produced as human and macaque IgG1 and IgG2. The anti-influenza mAb CH65 (21) was used as a negative control. As expected, ADCP activity was greater for immune complexes formed with IgG1 compared to IgG2 (**Figure 1A**). We did not observe significant differences when comparing ADCP activities of monocytes with the H^131^ and R^131^ allelic variants (Mann Whitney p>0.05) for any of the immune complexes tested (**Figure 1A**), across a range of mAb concentrations (**Supplementary Figure S2**). However, ADCP activity trended slightly higher for the H^131^ allele at the highest concentration of IgG2, more so for macaque than human. More specifically, the phagocytosis score was 6.6 ± 5 (mean ± standard deviation) and 1.6 ± 0.5 for human IgG1 and IgG2 and 11.5 ± 9.6 and 2.2 ± 0.9 for macaque IgG1 and IgG2 for (H/H) versus 5.6 ± 4.9 and 1.2 ± 0.26 for human IgG1 and IgG2 and 6.9 ± 5.1 and 1.3 ± 0.45 for macaque IgG1 and IgG2 for (R/R), respectively. These results are consistent with our and others prior work demonstrating that factors other than Fc receptor genetic variation influence heterogeneity in monocyte ADCP responses (15, 22–24), but could also reflect the small sample size and high variability in this assay.

To evaluate the effect of the FcγRIIa polymorphism on IgG subclass binding we measured the affinities of both human (H^131^ and R^131^) and rhesus macaque (H^131^ and P^131^) FcγRIIa variants and human FcγRIIb to a panel of IgG1–4 antibodies. Antibodies of both species were derived from the broadly neutralizing antibody VRC01, thus shared the same antigen specificity, allowing subclass-specific and species-specific comparisons. Human IgGs were expressed in their native form, while macaque IgGs were engineered as chimeras consisting of the human VRC01 variable region (V_H_1 and C_H_1) fused to macaque C_H_2 and C_H_3 domains, paired with the human VRC01 kappa light chain (V_K_1 and C_K_1). To assess both intra- and interspecies interactions, each IgG subclass was tested against both FcγRIIa variants from both species, e.g. human IgG1 was tested against macaque FcγRIIa(P^131^) and FcγRIIa(H^131^) in addition to human FcγRIIa(R^131^) and FcγRIIa(H^131^). Human IgGs were also tested against FcγRIIb (**Figure 1B**; **Supplementary Figure S3**-**S7**; **Supplementary Table S1**).

As previously reported (11), human IgG1 and IgG3 exhibited the highest binding affinities among all IgG subclasses and showed comparable average affinities to both the high- and low-affinity alleles of human FcγRIIa and human FcγRIIb (K_D_ values in the range of 6-14 µM) (**Figure 1B**; **Supplementary Table S1**). Human IgG2 did not show a clear preference for either FcγRIIa allele (K_D_ values of 10 ± 3 µM for R^131^ allele and 11 ± 3 µM for the H^131^ allele, mean ± standard deviation) but did bind poorly to FcγRIIb which also has R^131^ (K_D_ value of 30 µM). In contrast, IgG4 showed higher affinity for the R^131^ variant (K_D_ value of 15 µM versus a K_D_ of 44 µM for the H^131^ variant) and for FcγRIIb (K_D_ value of 9 µM). In general, we observed greater variability in binding measurements for human FcγRIIa(R^131^) compared to human FcγRIIa(H^131^) across human IgG subclasses, except for IgG4. These binding trends are largely consistent with our gel filtration results in making complexes for crystallization given that Fc generally binds better than full length IgG (25). We were able to make stable complexes of human FcγRIIa(H^131^) and IgG2 Fc but not of human FcγRIIa(H^131^) and IgG4 Fc or of human FcγRIIa(R^131^) and IgG2 Fc (data not shown). They are also largely consistent with reported values in the literature although we did see a smaller difference in IgG2 affinity between alleles than has been previously reported (26, 27).

In contrast to humans, all macaque IgG subclasses exhibited significantly higher binding affinities to the macaque high-affinity FcγRIIa(H^131^) allele (K_D_ values ranging from 8-21 µM), with relative affinities following in the order of IgG1>IgG2>IgG3>IgG4 (**Figure 1B**; **Supplementary Table S1**). However, in contrast to the mixed responses for the human IgG subclasses for the low affinity R^131^ variant, all macaque IgG subclasses displayed markedly reduced affinities for the low-affinity P^131^ variant with the K_D_s in the range of 26-102 µM. The lower affinity variant had affinities as much as 2–5 times lower that the high-affinity H^131^ variant consistent with other measurements of the macaque IgG subclasses to macaque FcγRIIa (12, 28).

In addition, we also evaluated species-mismatched binding interactions (**Figures 1A, B**; **Supplementary Table S1**). Human IgGs consistently showed significantly higher affinity for the H^131^ variant of the macaque FcγRIIa compared to the P^131^ variant (K_D_s in range of 5-18 µM and 33-99 µM for the H^131^ and P^131^ variants, respectively). Interestingly, among human IgG subclasses, IgG4 showed the least pronounced difference in the binding affinity to the high- and low-affinity macaque alleles, a roughly 2-fold difference versus a 7- to 8-fold difference for the other IgGs. In contrast, macaque IgGs exhibited relatively modest differences in binding to the human FcγRIIa variants, with K_D_ values in the range of 9-17 µM and 8-22 µM for the H^131^ and R^131^ alleles, respectively. A trend across species emerged consistent with previous reports (29); the lower-affinity FcγRIIa allele, R^131^ in humans and P^131^ in macaques, exhibited generally poorer binding to human or macaque IgG2. In macaques, this trend extended to all IgG subclasses from human or macaque which exhibited generally poorer binding for the P^131^ variant. These findings emphasize the functional relevance of the residue 131 polymorphism in modulating FcγRIIa-driven effector functions.

### 3.2 Structural insights into the FcγRIIa polymorphism and subclass-specific interactions reveal conserved and distinct features of the Fc–receptor interface across species

To elucidate the molecular basis underlying the observed differences in IgG subclass binding affinities and variant-specific functional responses, we employed structural biology using X-ray crystallography. We generated complexes of species matched IgG1 Fc with both the high- and low-affinity FcγRIIa variants from human and macaque. Additionally, we were able to form stable complexes of species matched IgG2 Fc with the high-affinity FcγRIIa variant in both species. We were able to successfully obtain crystals of each complex. We were also able to crystallize human IgG1 Fc with human FcγRIIb. Crystals of human FcγRIIa(H^131^) and FcγRIIa(R^131^) in complex with human IgG1 Fc diffracted to 2.38 Å and 2.85 Å, crystals of macaque FcγRIIa(H^131^) and FcγRIIa(P^131^) with macaque IgG1 Fc to 3.55 Å and 2.65 Å, and crystals of macaque FcγRIIa(H^131^) with macaque IgG2 Fc and of human FcγRIIa(H^131^) with human IgG1 Fc to 3.2 Å and 2.0 Å, respectively. Crystals of human FcγRIIb in complex with human IgG1 Fc diffracted to 3.07 Å. All crystals except for the human FcγRIIa(R^131^) in complex with IgG1 Fc contained one complex in the asymmetric unit, i.e. one receptor in complex with one Fc dimer; the human FcγRIIa(R^131^)-IgG1 Fc crystals contained two complexes in the asymmetric unit. Complete data collection and model statistics can be found in **Supplementary Table S2**.

**Figure 2:**
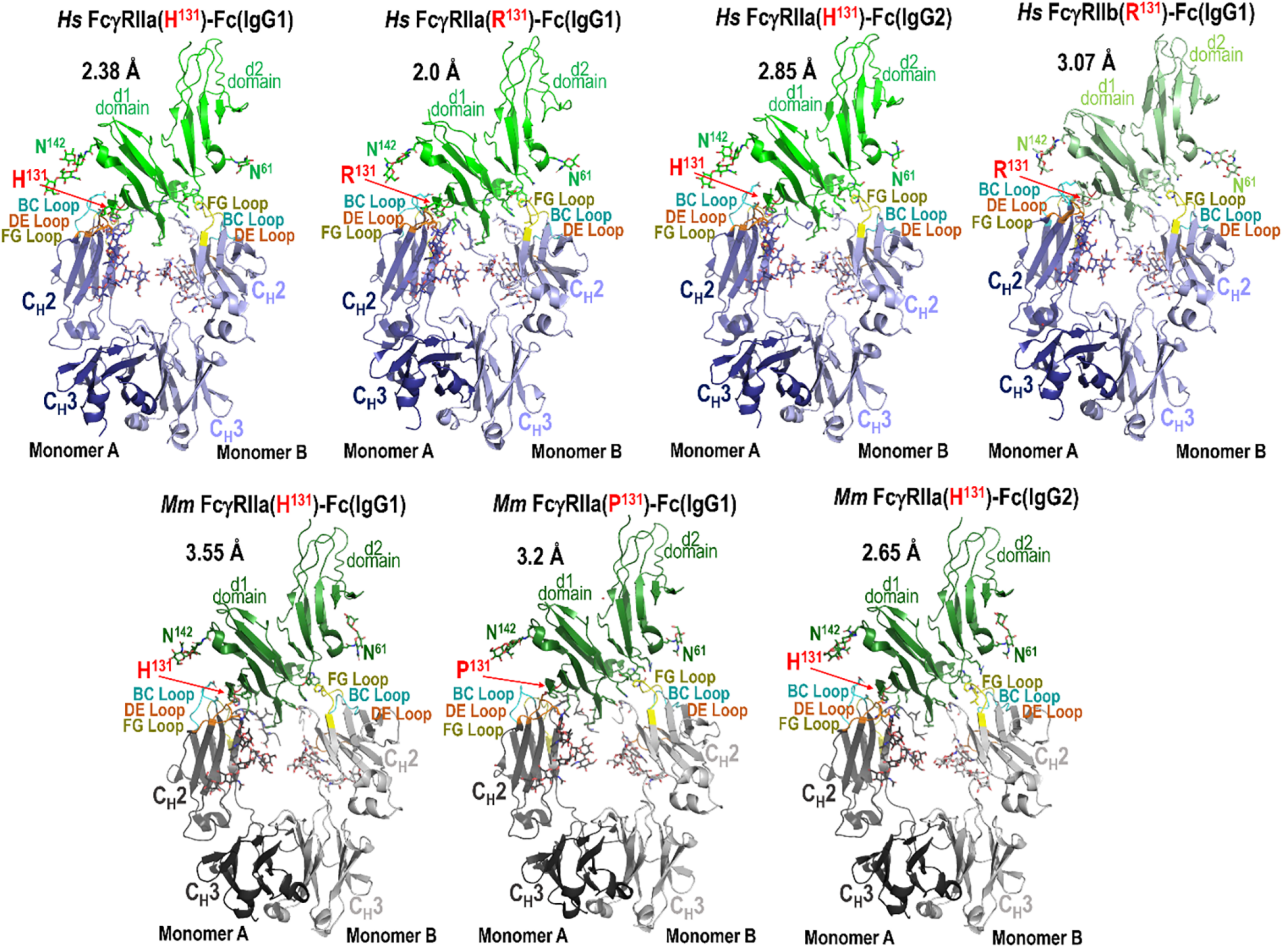
Crystal structures of the human and macaque FcγRIIa high and low affinity variants in complex with the Fc of species matched IgG1 or IgG2 and human FcγRIIb in complex with human IgG1 Fc. Structures were solved by X-ray crystallography at a resolution range of 2.65-3.55 Å for macaque and 2.0-3.07 Å for human. Complexes are shown as ribbons with balls and sticks for key residues and glycans. Receptors are colored green (human FcγRIIa), lighter green (human FcγRIIb), and darker green (macaque FcγRIIa). Fcs are colored blue (human) and gray (macaque) in darker or lighter color for monomer A or B respectively. Fc binding loops are colored as labeled.

**Figure 2** illustrates these structures, with Fc loops that contribute to the Fc-FcγRIIa/b interface highlighted as indicated, and the polymorphic residue at position 131 shown in red; human FcγRIIb, like human FcγRIIa(R^131^), has arginine at position 131. Consistent with previously reported FcγR-Fc structures, all FcγRIIa/b-Fc complexes exhibit an asymmetric binding mode, in which each monomer of the Fc dimer engages distinct regions of the receptor. For clarity and consistency, the two Fc protomers will be referred to as monomer A and monomer B throughout this analysis. This designation aligns with chain labeling conventions used in the majority of Fcγ-Fc complex structures deposited in the Protein Data Bank (PDB). In these complexes, monomer A of the Fc mainly interacts with the d1 domain of FcγRIIa/b, where residue 131 is located (H/R^131^ in humans or H/P^131^ in macaques), while monomer B mainly interacts with the d2 domain of FcγRIIa/b and the hinge region between the two domains. Structural alignments indicate that, while the interaction between monomer B and the receptor is largely conserved, aside from sequence differences in the receptor and Fc region between the two species, the interactions involving monomer A vary depending on the IgG subtype, receptor variant, and species. The most pronounced differences occur at the N-terminus of the Fc, in the lower hinge region, and the molecular details of these interactions provide a structural basis for the observed affinity differences between receptors and IgG subtypes.

### 3.3 Fc monomer B interactions with FcγRIIa/b are highly conserved with subtle species-specific and receptor-specific differences

**Figure 3:**
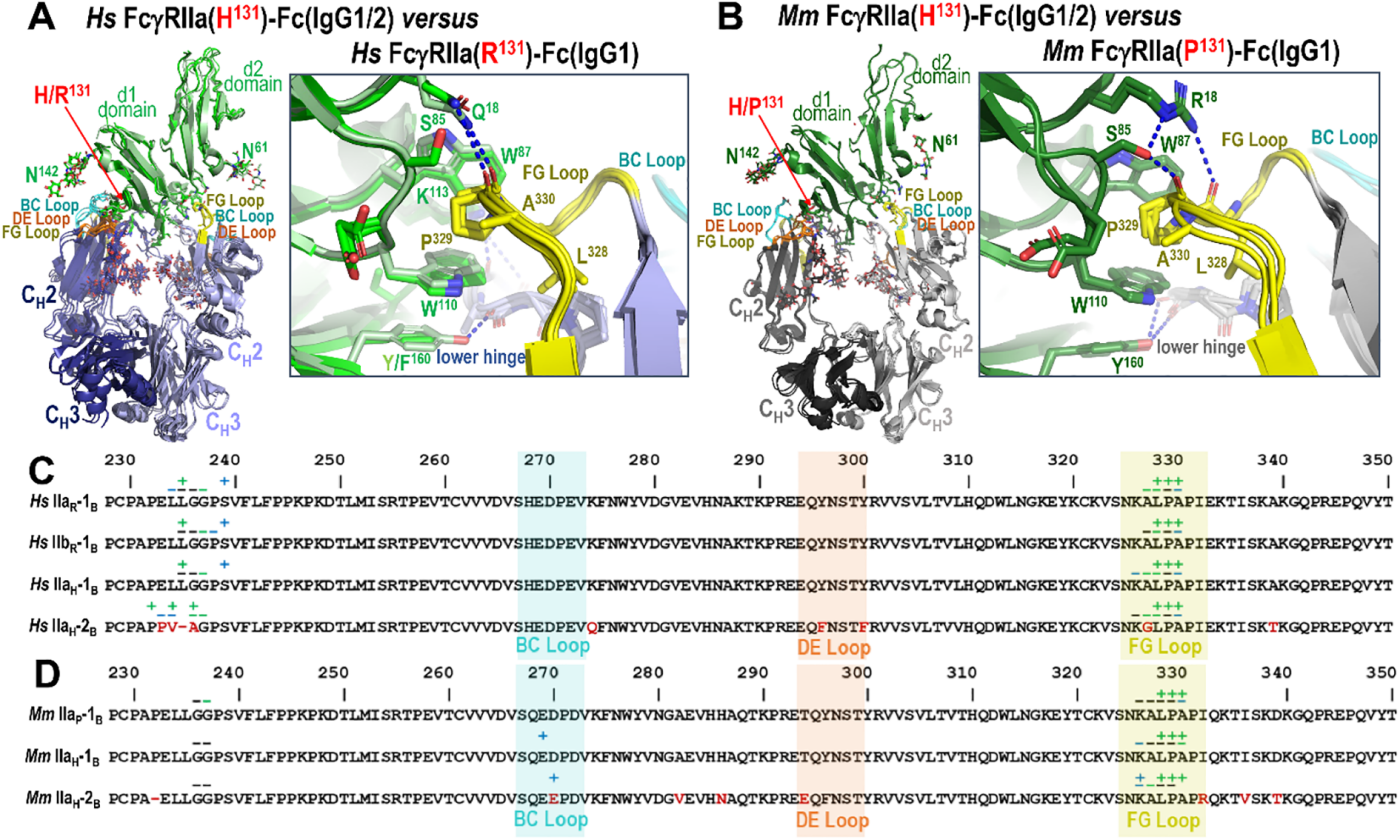
Comparison of Fc monomer B-FcγRIIa/b receptor contacts between the human H/R^131^ and the macaque H/P^131^ allelic variant pairs. **(A, B)** Structural superposition of the human FcγRIIa and FcγRIIb complexes with an expanded view into the human Fc monomer B interface **(A)** and structural superposition of the macaque FcγRIIa complexes with an expanded view into the macaque Fc monomer B interface **(B)**. Colors are as in **Figure 2**. **(C, D)** The Fc monomer B residues contributing to human FcγRIIa and FcγRIIb binding **(C)** and the Fc monomer B residues contributing to macaque FcγRIIa receptor binding **(D)**. Fc monomer B contact residues defined by a 5 Å cutoff are marked above the sequence with (+) for side chain and (-) for main chain to indicate the type of contact. Contact types are colored as follows: hydrophilic (blue), hydrophobic (green) and both (black). Loop regions are as indicated. Sequence differences in IgG2 relative to IgG1 are shown in red.

In both species, monomer B of either IgG1 or IgG2 engages FcγRIIa primarily through two regions of the Fc: the FG loop (between the F and G β-strands) and the lower hinge region (**Figure 3**). In both species the defining feature of the monomer B interface is the sandwich of FG loop proline P^329^ between receptor tryptophans W^87^ and W^110^. In the human complex, additional stabilization is provided by receptor residues Q^18^, which can also form an H-bond to the carbonyl of P^329^ of the Fc, and K^113^, which can form an H-bond to the carbonyl of residue 234 or 235 in the lower hinge region (**Figures 3A, C**; **Supplementary Figure S8**). In contrast, in macaques the analogous interactions differ slightly because of differences in receptor sequence, i.e. receptor residue R^18^ forms an H-bond to the carbonyl of A^330^ in the FG loop and residue Y^160^ forms an H-bond to the carbonyl of G^236^ in the lower hinge (**Figures 3B, D**; **Supplementary Figure S8**). Human FcγRIIa F^160^ is unable to form an H-bond but instead permits a tighter more hydrophobic interaction than macaque FcγRIIa Y^160^, which shifts the lower hinge approximately 0.5 Å further away. It should be noted that human FcγRIIb also has Y^160^ which forms an H-bond to the carbonyl of G^236^ similar to macaque FcγRIIa (**Figures 3A, C**; **Supplementary Figure S8**). On the whole, the overall architecture of the interface is well-conserved across both variants for both species, however there are still some individual contacts that vary slightly between structures due to differences in resolution and conformational flexibility.

Despite this similarity, we observed modest but consistent species-specific differences in buried surface area (BSA) contributed by monomer B. In human complexes, monomer B contributes 38.9-43.7% of the total Fc dimer BSA, compared to 38.1-40.1% in macaques (**Supplementary Tables S3**, **S4**). Total Fc BSA values are also higher in human complexes [896 Å^2^ for FcγRIIa(H^131^)-IgG1 Fc, 950 Å^2^ for FcγRIIa(H^131^)-IgG2 Fc, 998 Å^2^ for FcγRIIa(R^131^)-IgG1 Fc, and 1031 Å^2^ for FcγRIIb-IgG1 Fc] than in macaque complexes [802 Å^2^ for FcγRIIa(H^131^)-IgG1 Fc, 831 Å^2^ for FcγRIIa(H^131^)-IgG2 Fc, and 838 Å^2^ for FcγRIIa(P^131^)-IgG1 Fc]. This difference is largely attributable to reduced contributions from the lower hinge region in macaque monomer B [e.g., 85-93 Å^2^ in macaques vs. 181-223 Å^2^ in humans] and may reflect the lower affinity of the macaque receptors for IgG as compared to human or the generally lower resolution of the macaque complex data (2.65-3.55 Å for macaque and 2.0-3.07 Å for human). Thus, although the general binding mode of monomer B is conserved, specific interactions in the lower hinge region differ between species and receptor type and contribute to subtly altered receptor engagement.

### 3.4 The Fc monomer A FcγRIIa interactions strictly depend on the residue at position 131 in a species- and subclass-specific manner

**Figure 4:**
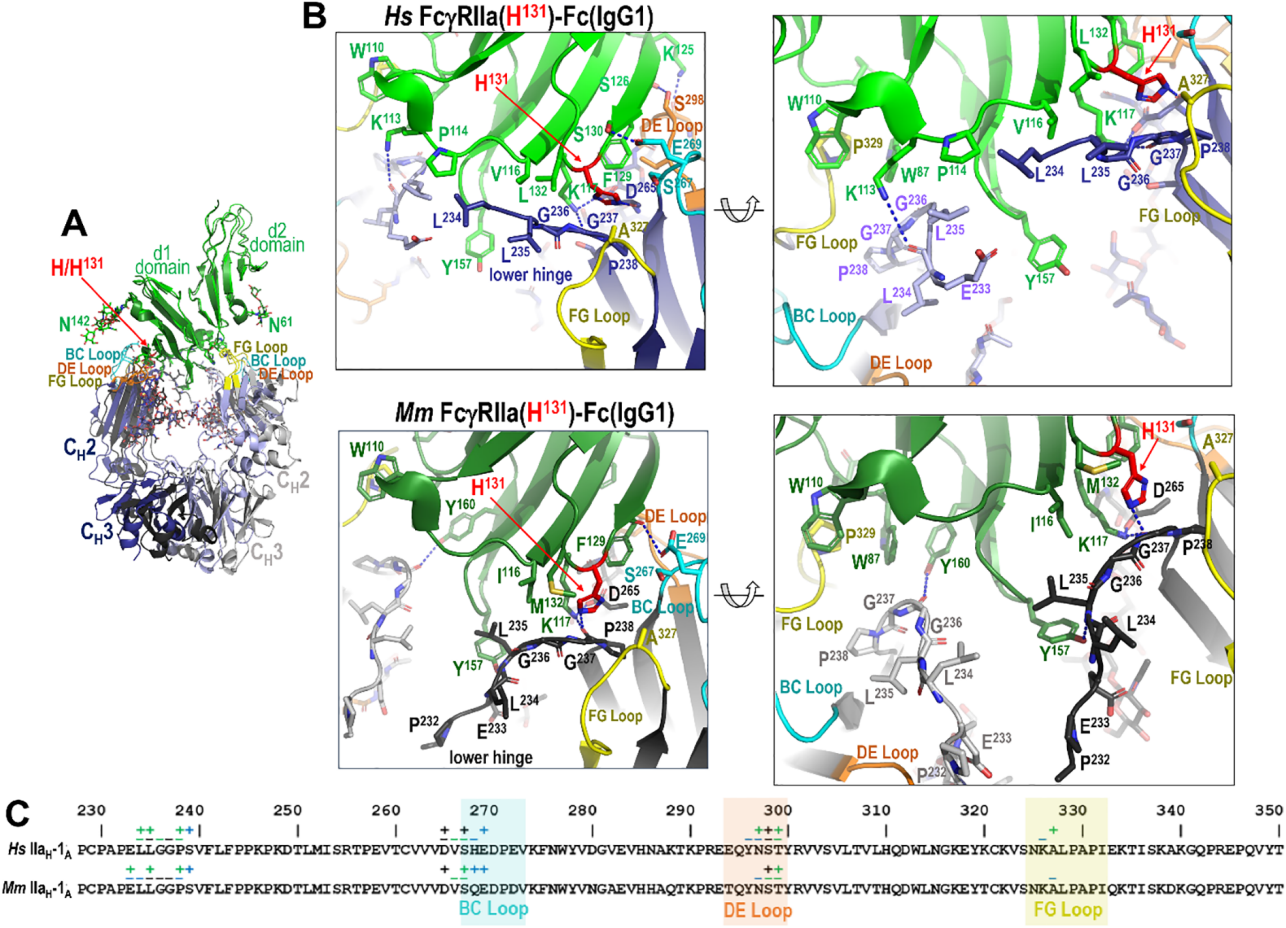
The IgG1 Fc-FcγRIIa interface in the human and macaque high affinity H^131^ alleles. **(A)** Receptor based superimposition of the human and macaque FcγRIIa(H^131^) IgG1 Fc complexes. Colors are as in **Figure 2**. **(B)** Magnified views into the IgG1 Fc-receptor interface, human top and macaque bottom, with residues contributing to the interface shown as sticks and H-bonds shown as blue dotted lines. Two alternate views for each complex are shown (the right panel is rotated approximately 30°from the left). **(C)** The Fc monomer A residues contributing to FcγRIIa receptor binding mapped onto the Fc primary sequence with contacts defined as in **Figure 3**. H^131^ in the high affinity allele of both species forms a network of interactions with residues of the lower hinge region of monomer A.

**Figure 5:**
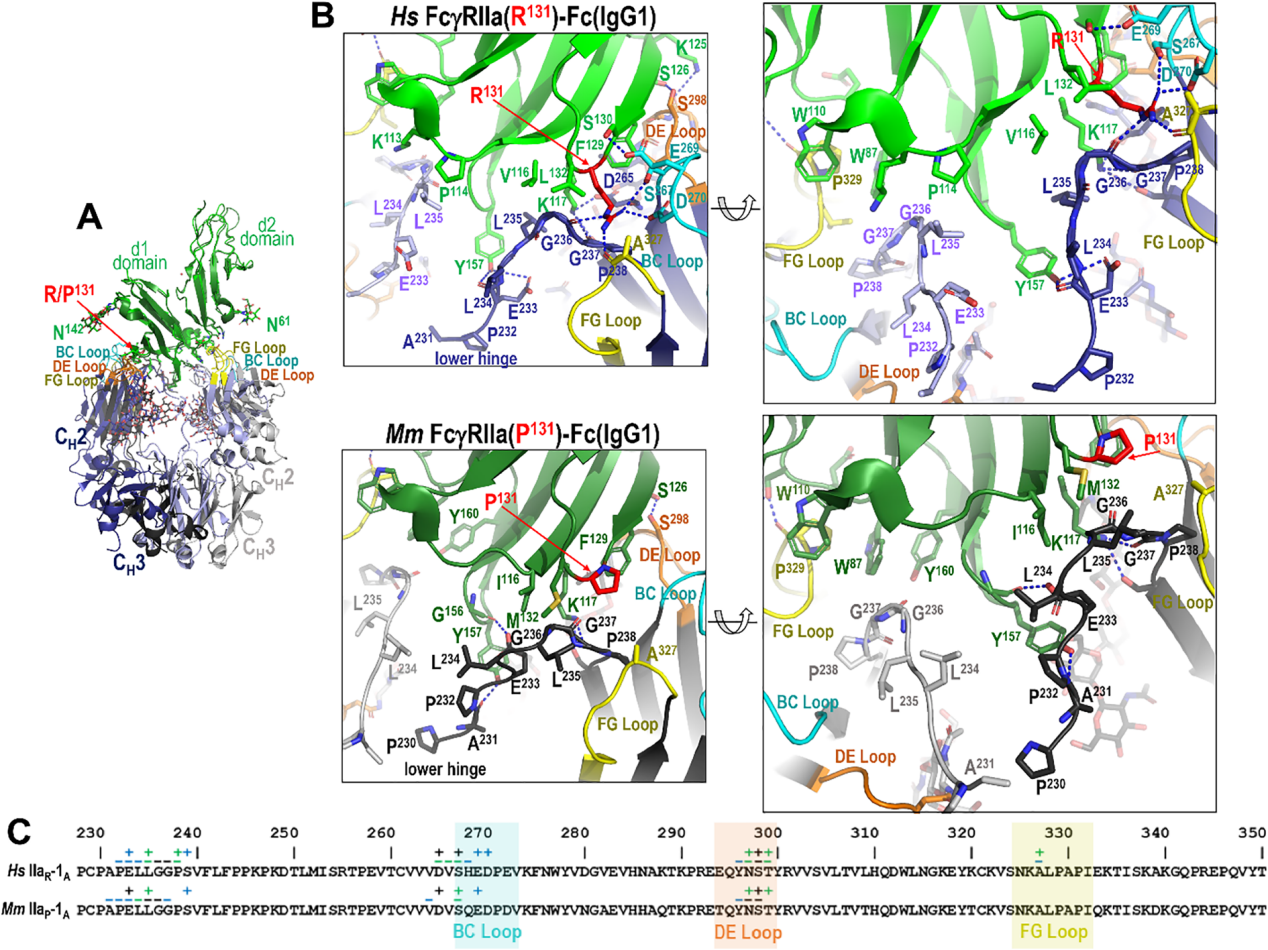
The IgG1 Fc-FcγRIIa interface in the human and macaque low affinity R/P^131^ allele. **(A)** Receptor based superimposition of the human FcγRIIa(R^131^)-Fc(IgG1) and macaque FcγRIIa(P^131^) IgG1 Fc complexes. Colors are as in **Figure 2**. **(B)** Magnified views into IgG1 Fc-receptor interface, human top and macaque bottom, with residues contributing to the interface shown as sticks and H-bonds shown as blue dotted lines. Two alternate views for each complex are shown (the right panel is rotated approximately 30°from the left). **(C)** The Fc monomer A residues contributing to FcγRIIa receptor binding mapped onto the Fc primary sequence with contacts defined as in **Figure 3**. R^131^ in the low affinity allele of the human complex forms a network of interactions with residues of the lower hinge of monomer A, but P^131^ in the macaque complex does not.

**Figure 6:**
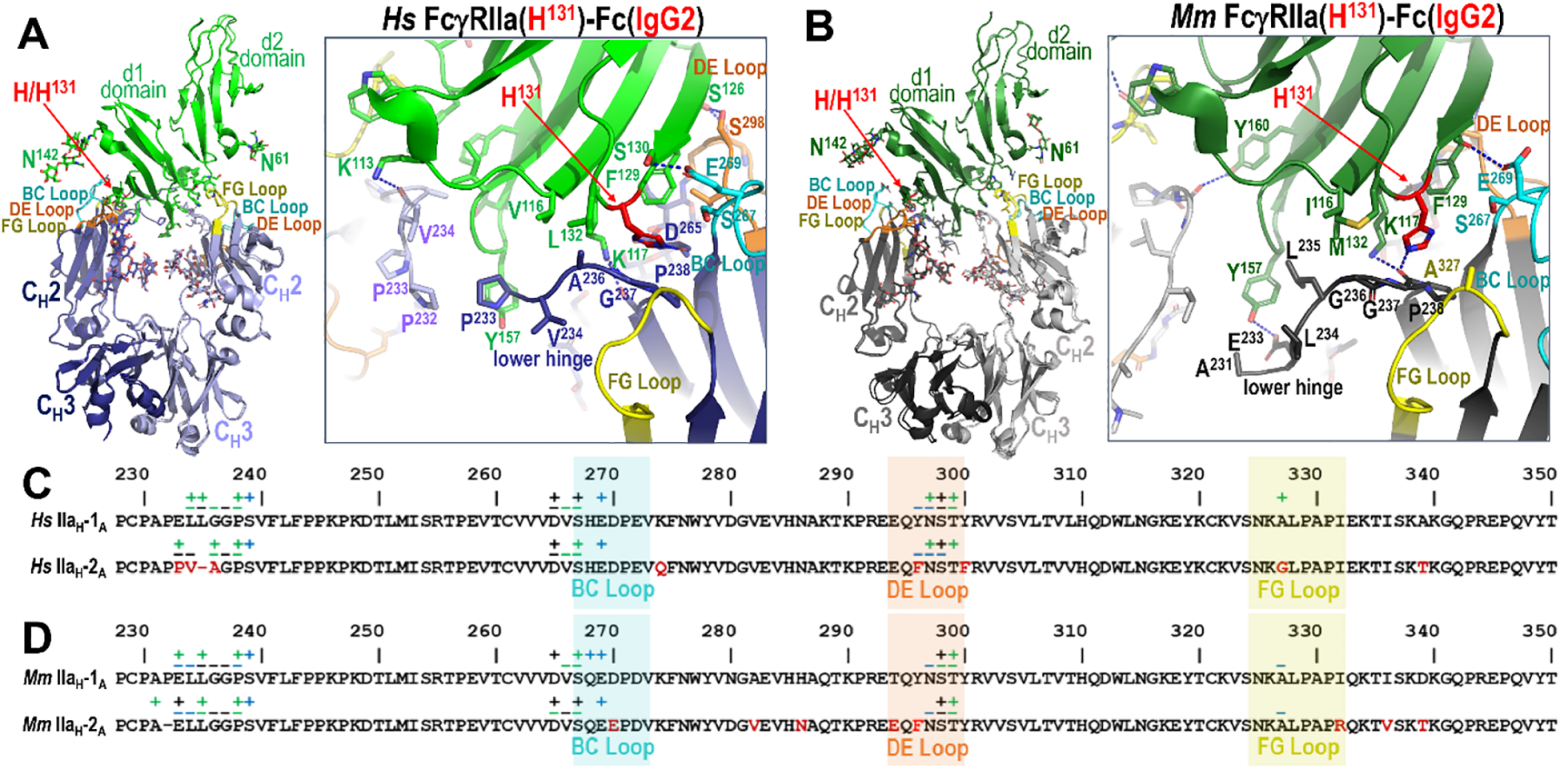
IgG1 versus IgG2 binding to the FcγRIIa H^131^ allele in human and rhesus macaque. **(A, B)** Receptor based superimposition of the human FcγRIIa(H^131^) IgG1 and IgG2 complexes **(A)** and the macaque FcγRIIa(H^131^) IgG1 and IgG2 complexes **(B)** with expanded views into the interface. Residue labeling and the view orientations are as in **Figure 4** and **5**. **(C, D)**. The Fc monomer A residues contributing to human FcγRIIa IgG1 and IgG2 receptor binding **(C)** and to macaque FcγRIIa IgG1 and IgG2 receptor binding **(D)** mapped onto the primary Fc sequence with contacts defined as in **Figure 3**. Sequence differences in IgG2 relative to IgG1 are shown in red. In both species an extensive network of contacts stabilizes the Fc-FcγRIIa interface, but the IgG2 Fc-FcγRIIa interface has fewer contacts due to an amino acid deletion in the lower hinge region of IgG2. Amino acid changes in human IgG2 in the region surrounding the deletion further modify the interaction with the lower hinge in the human complex.

In contrast to monomer B of Fc, monomer A displays more variability in its interaction with FcγRIIa, with differences that reflect both species-specific receptor sequence and IgG subclass-dependent Fc architecture. This is mostly driven by the polymorphism at residue 131 which dictates how the lower hinge interacts with the receptor. As shown in **Figures 4**, **5**, **6** for the high- and low-affinity FcγRIIa variant Fc complexes, the BC and DE loops in combination with lower hinge anchor monomer A to the receptor.

Most of these interactions, i.e. those from or near the BC and DE loops, are largely conserved across species and IgG subclasses. More specifically, Fc residue D^265^ immediately preceding the BC loop and E^269^ from BC loop form stable hydrogen bonds with receptor K^117^ and Ser^130^, respectively. The other BC loop residues largely contribute hydrophobic van der Waals contacts to the receptor. Exceptions are seen in the human FcγRIIa(R^131^)-IgG1 Fc complex, where R^131^ can also form an hydrogen bond with D^270^ (**Figures 4B, C**), and in the macaque FcγRIIa(P^131^)-IgG1 Fc complex, where E^269^ engages in only a very weak hydrogen bond to the carbonyl oxygen of F^129^ (**Figures 5B, C**). DE loop interactions, on the other hand, are mainly hydrophobic although a hydrogen bond between Fc S^298^ and receptor S^126^ or the carbonyl oxygen of Fc Y^296^ and receptor S^126^ is formed in most or some complexes, respectively. More specifically, the S^298^-S^126^ contact is hydrophobic instead of hydrophilic in the two macaque FcγRIIa(H^131^) complexes and a Y^296^ carbonyl oxygen-S^126^ hydroxyl hydrogen bond is only seen in the human FcγRIIa(R^131^) and macaque FcγRIIa(P^131^) complexes. It should be noted that the two macaque FcγRIIa(H^131^) complexes were determined at generally lower resolution, 3.55 Å for IgG1 Fc and 3.2 Å for IgG2 Fc, than the other structures reported here and are thus subject to a lower level of certainty.

The lower hinge region, in contrast, plays an important role in IgG subclass- and polymorphism-specific binding as it is stabilized in part by the residue at position 131. In the high affinity allele of both species, H^131^ and K^117^ help stabilize the lower hinge allowing it to pack against P^114^, V^116^ and L^132^ in humans or I^116^ and M^132^ in macaques (**Figures 4B, C**). In the low affinity R^131^ allele in humans, stabilization persists but is slightly weakened, e.g. R^131^and K^117^ stabilize the lower hinge but packing against P^114^, V^116^ and L^132^ is reduced by the shift of the lower hinge away from V^116^ and toward Y^157^ (**Figures 5B, C**). In macaques, however, P^131^ fails to contribute meaningful stabilization, leaving K^117^ as the sole residue anchoring the lower hinge, with little contribution from P^131^, I^116^ or M^132^. This results in a looser, more variable conformation in the low-affinity macaque complexes. Given that the most prominent sequence differences between IgG1 and IgG2 lie in the lower hinge region in both species, this structural variation likely underpins the observed subclass-specific affinity differences across species with the caveat that the macaque IgG1 and IgG2 complexes were determined to a generally lower resolution than the other complexes in this paper. Taken together, these results emphasize that monomer A provides a structurally dynamic interface shaped by both receptor polymorphism and IgG subclass, with distinct patterns between humans and macaques.

### 3.5 The lower hinge region drives the allele-specific receptor interactions of IgG2 in humans

Human IgG2 differs only modestly from IgG1 in its Fc region, with 95.5% sequence identity. However, three amino acid substitutions occur at the receptor-binding interface in the BC, DE, and FG loops: Y^296^ and Y^300^ become F^296^ and F^300^ in the DE loop and A^327^ becomes G^327^ in the FG loop in IgG2 (**Figure 6**). Among these, only A^327^ in IgG1 makes meaningful contact with FcγRIIa through van der Waals interactions involving its β-carbon, an interaction that is lost in IgG2 due to the substitution of glycine (**Figure 6A**). Neither Y^300^ nor F^300^ are contact residues, and Y^296^ and F^296^ only contribute van der Waals contacts through main chain atoms and their α-carbon which are identical for both residues. Thus, we assume that most of the difference in this region is due to the alanine to glycine change at position 327 in IgG2. While we were only able to determine the complex with IgG2 for the His^131^ variant, we expect that there would be a similar loss in the IgG2 FcγRIIa(R^131^) variant complex since the α-carbon of A^327^ contributes similarly to the IgG1 FcγRIIa(R^131^) complex interface (**Figure 6A**). These subtle sequence changes in the DE and FG loops help explain the relatively modest reduction in IgG2 affinity for human FcγRIIa but are unlikely to fully account for the more pronounced allele-specific differences observed functionally.

The most significant differences between IgG1 and IgG2 in human lie in the IgG2 hinge, which is shorter and more rigid than IgG1 with up to four interchain disulfide bonds (4), and in the lower hinge region, where IgG2 features a one-residue deletion and three amino acid change in sequence in residues surrounding the deletion. Only the lower hinge directly interacts with FcγRIIa. This deletion effectively eliminates the contributions to the interface in the FcγRIIa(H^131^)-IgG1 Fc complex from L^234^ and L^235^ in the FcγRIIa(H^131^)-IgG2 Fc complex. Instead, A^236^ replaces G^236^ in the IgG2 complex which packs against L^132^ and V^116^ of the receptor less well than L^235^ in the IgG1 complex. To partially compensate for the loss of L^235^-mediated contacts, IgG2 Fc residues V^234^ and P^233^ shift towards receptor Y^157^ to allow π-π stacking between P^233^ and the aromatic ring of Y^157^, an interaction not observed in IgG1; IgG1 has E^233^ instead of P^233^. However, this interaction would likely be less stable in the R^131^ variant, because the orientation of the lower hinge is different when interacting with R^131^ as compared to H^131^. This potentially explains the observed affinity difference for the two alleles to IgG2.

Altogether, these findings suggest that structural changes in the lower hinge, particularly in IgG2, contribute significantly to both subclass- and allele-specific differences in receptor binding in human. They also reinforce the idea that even minor sequence alterations can affect FcγR interactions in a context-dependent manner, especially in the polymorphic and functionally divergent FcγRIIa receptor.

### 3.6 The deletion in the lower hinge region of macaque IgG2 disrupts key contacts and reduces affinity for the P^131^ allele

Most of the differences between macaque IgG2 and macaque IgG1 similar to its human counterpart reside in the hinge region, which is shorter in macaque IgG2. Macaque IgG2 also has a deletion in the lower hinge region as compared to macaque IgG1 (**Figures 6C, D**). The Fc domains of macaque IgG1 and IgG2 are also highly conserved, sharing 93.4% sequence identity. Differences between the two subclasses in macaques are limited to four residues located within the BC, DE, and FG loops: D^270^ in IgG1 is E^270^ in IgG2, T^294^ in IgG1 is E^294^ in IgG2, Y^296^ in IgG1 is F^296^ in IgG2, and I^332^ in IgG1 is R^332^ in IgG2 (**Figure 6D**).

Of these, only Y^296^ contributes to the Fc-FcγR interface in any of the macaque complexes. This interaction occurs exclusively in the FcγRIIa(P^131^)-IgG1 Fc complex through main chain atoms that are not dependent upon the amino acid type, rendering the Y to F substitution in IgG2 functionally irrelevant. Consequently, the observed difference in affinity for IgG1 versus IgG2 in macaque solely resides in residues in the lower hinge region, i.e. the deletion of P^232^ in IgG2. However, comparison of the two FcγRIIa(H^131^) macaque complexes for IgG1 and IgG2 reveal that this deletion has only a modest structural impact (**Figure 6B**). The disruption results in only a modest reduction in receptor engagement for the H^131^ variant. Specifically, in the FcγRIIa(H^131^)-IgG1 Fc complex the carbonyl oxygen of E^233^ forms a strong hydrogen bond with the hydroxyl group of receptor Y^157^, and the aliphatic regions of E^233^ and L^235^ form favorable packing interactions with Y^157^. Additionally, L^235^ engages in hydrophobic van der Waals contacts with I^116^ of the receptor. On the other hand, in the FcγRIIa(H^131^)-IgG2 Fc complex, while these packing interactions are largely preserved, the hydrogen bond to Y^157^ is lost due to the altered position of E^233^ because of the P^232^ deletion. The aliphatic portions of E^233^ and L^235^ pack against the aromatic ring of Y^157^ and L^235^ packs against I^116^. Therefore, the one amino acid deletion in IgG2 seems to mainly disrupt the hydrogen bond to Y^157^, again with the caveat that the macaque IgG1 and IgG2 complexes were determined to a generally lower resolution than the other complexes in this paper.

In the FcγRIIa(P^131^)-IgG1 Fc complex, the carbonyl oxygen of A^231^ forms a strong hydrogen bond to the hydroxyl of receptor Y^157^and P^232^ packs against the aromatic ring of receptor Y^157^. This can be attributed to a shift in how the lower hinge binds FcγRIIa(P^131^) as compared to FcγRIIa(H^131^) (**Figures 4B**, **5B**). Therefore, the deletion of P^232^ in IgG2 likely disrupts the hydrogen bond to the hydroxyl of Y^157^ as well as reduces van der Waals contacts mediated by P^232^; a more conformationally constrained P^230^ takes the place of A^231^ and a less hydrophobic A^231^ takes the place of P^232^ in IgG2. Thus, the difference in the lower hinge conformation potentially accounts for the more pronounced reduction in affinity for IgG2 to FcγRIIa(P^131^) as compared to FcγRIIa(H^131^).

Overall, structural comparisons of IgG1 and IgG2 complexes with macaque FcγRIIa variants suggest that the P^232^ deletion in macaque IgG2 critically impairs receptor engagement, particularly in the context of the P^131^ allele. These differences likely translate into reduced functional responses, including impaired FcγRIIa-mediated effector functions such ADCP, and should be carefully considered in the design and interpretation of non-human primate immunogenicity studies.

### 3.7 The human FcγRIIa(R^131^) complex serves as a blueprint for understanding human FcγRIIb affinity differences

**Figure 7:**
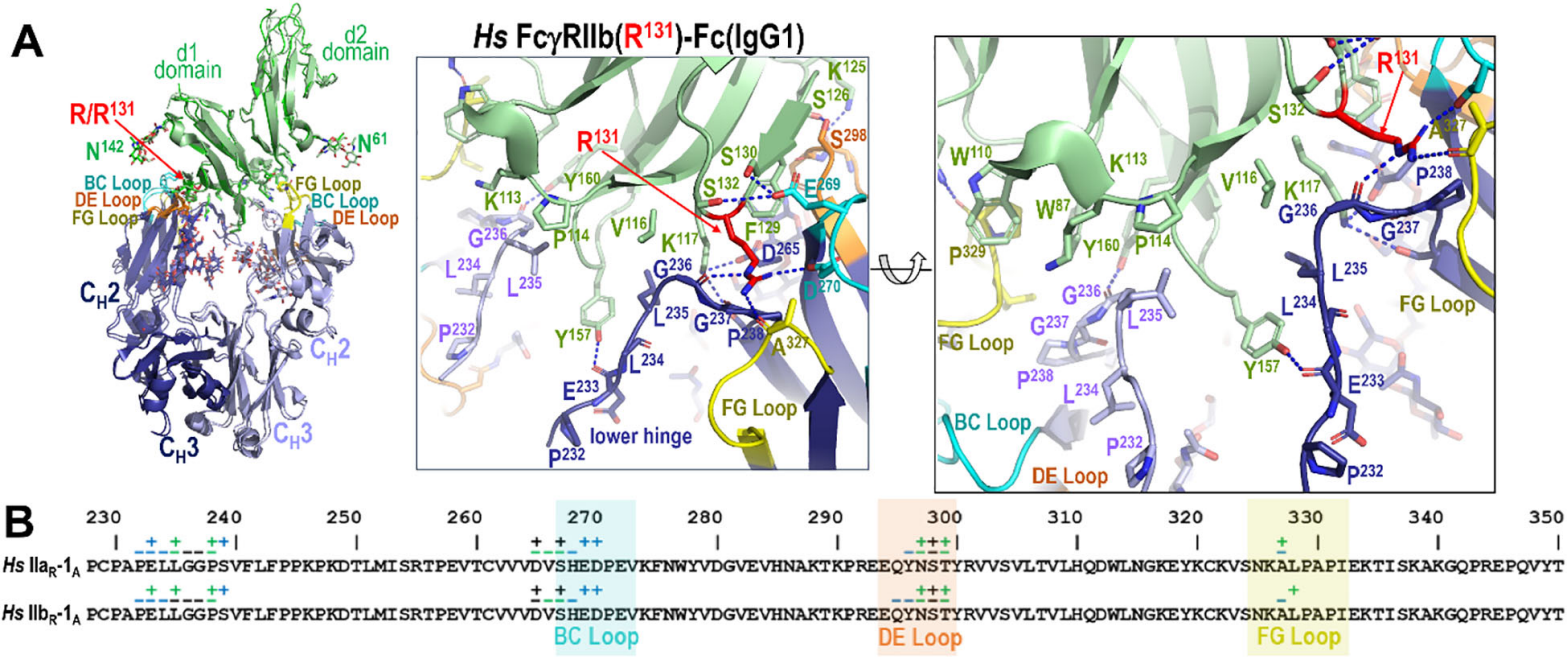
IgG1 Fc binding to FcγRIIb in human. **(A)** Receptor based superimposition of the human FcγRIIb and human FcγRIIa(R^131^) IgG1 Fc complexes with a magnified view of the human FcγRIIb complex. The residue labeling and the view orientations are as in **Figure 5**. **(B)** The Fc residues contributing to FcγRIIa and FcγRIIb receptor binding mapped onto the Fc primary sequence with contacts defined as in **Figure 3**. R^131^ in the low affinity allele of human FcγRIIa plays a similar role to R^131^ in human FcγRIIb.

The extracellular domain of human FcγRIIb is 94.2% and 93.6% identical in sequence to the extracellular domains of the FcγRIIa(R^131^) and FcγRIIa(H^131^) variants, respectively, due to the residue at position 131 which is arginine in FcγRIIb (**Supplementary Figure S8**). This contrasts with macaques, which have His at position 131 in FcγRIIb. We were able to confirm this close structural similarity with our structure of FcγRIIb in complex with IgG1 Fc (**Figure 7**). In total, there are only three residue changes in FcγRIIb relative to FcγRIIb(R^131^) that are part of the Fc-FcγRIIb interface. The first residue change at position 127, Q^127^ in FcγRIIa and K^127^ in FcγRIIb, is only involved in the interface via main chain atoms that are not dependent on residue type. The second residue change, as was discussed earlier, is the Y^160^ change in FcγRIIb versus the F^160^ in human FcγRIIa. Y^160^ shifts the lower hinge of monomer B approximately 0.5 Å further from the receptor and adds a hydrogen bond as compared to F^160^ in human FcγRIIa. This makes the interface more hydrophilic at the expense of hydrophobic van der Waals interactions. Specifically, the aromatic ring of F^160^ is approximately 4 Å away from G^236^ in both human FcγRIIa complexes, while the aromatic ring of Y^160^ is approximately 4.4 Å away from G^236^ in the human FcγRIIb complex. The third residue change is S^132^ in place of L^132^ in FcγRIIb. L^132^ in human FcγRIIa(H^131^) is important in stabilizing the lower hinge of monomer A by packing against Fc residue L^235^. L^132^ in human FcγRIIa(R^131^) is also important in stabilizing the lower hinge of monomer A but a shift in the lower hinge sequence places it against G^236^ instead of L^235^. The change to Ser in FcγRIIb leaves a gap and removes these hydrophobic van der Waals contacts. S^132^ instead forms a weak hydrogen bond to E^269^ in the BC loop; E^269^ is also involved in a stronger H-bond with S^130^ in FcγRIIb and in both FcγRIIa variants. Therefore, of the three changes in sequence only two potentially decrease affinity, the F^160^ to Y^160^ change affecting binding to Fc monomer B and the L^132^ to S^132^ change affecting binding to Fc monomer A. Interestingly both of these changes have an analogous change in macaque FcγRIIa; macaque FcγRIIa has Y^160^ in place of F^160^ and has M^132^ in place of L^132^, supporting the observation that residue changes at these two positions modulate Fc receptor affinity.

## 4 Discussion

Previous research on Fcγ receptors has predominantly centered on FcγRIIIa, which is known for its unique ability to have its IgG-binding affinity modulated greatly by glycan composition, both on the receptor and within the Fc region (30–32). In particular, afucosylated glycans on the IgG can increase affinity to the receptor from the micromolar to the nanomolar range (33). This has increasingly led to the use of afucosylated IgGs as therapeutic antibodies to maximize FcγRIIIa mediated effector functions (34).

In contrast, FcγRIIa has received comparatively less attention. Despite the high degree of structural similarity in their extracellular domains, FcγRIIa and FcγRIIIa share only about 44% sequence identity. Importantly, FcγRIIa in both human and Rhesus macaque also lack a glycan at the equivalent position of N^162^ in FcγRIIIa, one of the key sites implicated in affinity enhancement for afucosylated IgG (33). Consistent with the limited effect of specific glycotype on binding affinity reported previously (35, 36), our studies using the allelic variants of FcγRIIa from both human and macaque show limited contact with the Fc glycan and show no glycan-glycan interactions with the Fc. This indicates that in both species FcγRIIa receptor engagement is less dependent on glycan modification as compared to its FcγRIIIa counterpart. Nevertheless, our structural studies were conducted using proteins with wild-type glycosylation produced in HEK293F cell lines. Previously we and others have shown that Fcs and IgGs from HEK293F cell lines display predominantly complex-type biantennary structures with high levels of processing including galactosylation, sialylation, and core fucosylation (37, 38). While there is some indication that glycan composition, specifically with respect to afucosylation, may also differentially impact FcγRIIa affinity (39), our IgGs did not show an effect.

Prior to our work, only a single structure of human FcγRIIa in complex with human Fc had been reported, the low-affinity variant FcγRIIa(R^131^) by Ramsland et al. (40). Interestingly, the human FcγRIIa(R^131^) complex structure presented here is more similar to the human high-affinity variant FcγRIIa(H^131^) complex structure we determined, than to the FcγRIIa(R^131^) complex determined previously (PDB ID 3RY6). The main difference between the two FcγRIIa(R^131^) complex structures is that the earlier structure is more open with a weaker association of monomer A of the Fc with the receptor. There is also a greater glycan contribution to the receptor-Fc interface in that structure (**Supplementary Table S4**). A difference in glycan composition could be one possible explanation for the differences we observe between the two structures. The FcγRIIa(R^131^) in 3RY6 was produced in insect cells while the FcγRIIa(R^131^) in our structure was produced in HEK 293 cells. Furthermore, our structure was also determined at a slightly higher resolution. For these reasons, all comparative analyses in this study were performed using the FcγRIIa(R^131^) structure reported here.

The two human allotypes have been described differently in the literature over time. Originally described as low responder (LR) and high responder (HR) allotypes based upon their affinity to mouse IgG1 (40), they have more recently been referred to as high affinity and low affinity variants based upon their relative affinity to human IgG, i.e. the FcγRIIa(H^131^) and FcγRIIa(R^131^) variants, respectively (28). We have used the latter definition in our reference to the two allotypes even though in our hands we did not always see a significant difference between the two. This was not the case for the macaque allotypes at position 131, which clearly showed lower affinity for the FcγRIIa(P^131^) allele for every IgG tested from both human and macaque. In humans this difference in affinity has been reported to be significant particularly with respect to IgG2. In our ADCP assay we did see a trend for higher activity for the H^131^ allele, but it was not significant. This is in contrast with other reported cell-based assays which have shown a clear preference for the H^131^ allele (28, 41). One possible explanation for this difference may come from the nature of the ADCP assay used whose signal can be dominated by hinge length (15, 29); IgG2 has the shortest hinge length of the four human IgGs. Any allele specific differences may have therefore been overshadowed by the effect of the shorter hinge in IgG2. IgG2 also has another complication, disulfide bond isomerization in its upper hinge (42, 43), which could also have had an impact on ADCP. Although we did not characterize the disulfide isoform distribution for our IgG2 sample, we did use the same IgG2 sample for both FcγRIIa variants which should have mitigated any differences due to disulfide bond isoform. Another caveat is that in cell-based assays the activating FcγRIIa is rarely present without inhibitory FcγRIIb, FcγRIIb with arginine at position 131 did show significantly lower affinity towards IgG2, and potentially other FcγRs. Thus, certain FcγRIIa/FcγRIIb combinations on top of a mixed FcγR repertoire may magnify the allele specific differences in affinity of FcγRIIa towards IgG2.

We were also able to confirm that the human FcγRIIa(R^131^) complex is almost identical to FcγRIIb and by extension FcγRIIc, as FcγRIIb and FcγRIIc are identical in their extracellular domains. There are essentially two discriminating residue changes that differentiate FcγRIIa(R^131^) from FcγRIIb. The first is the Y^160^ change from F^160^ in FcγRIIa. This adds a hydrogen bond to the FcγRIIb complex but shifts the lower hinge approximately 0.5 Å away from the receptor. A similar conformation is seen in macaque FcγRIIa which normally has Y^160^. The second is the change from L^132^ to S^132^ in FcγRIIb. L^132^ and V^116^ form a hydrophobic surface that helps stabilize the lower hinge. S^132^ in FcγRIIb removes much of this surface and replaces it only with a weak hydrogen bond to E^269^. Other structures of FcγRIIb were solved with mutated versions of the Fc designed to enhance FcγRIIb binding (44). Initial mutagenesis designed to enhance FcγRIIb binding over FcγRIIa focused on a P^238^D mutation that enhanced binding to Y^160^ (34). Later optimization added five other mutations to enhance discrimination from FcγRIIa(R^131^) (44). Both mutant Fc complexes with FcγRIIb are highly similar to our FcγRIIb complex structure (**Supplementary Table S4**); differences mainly center in the N-terminal regions of the Fc that are poorly resolved in those structures. Both structures are also more similar to our FcγRIIb and FcγRIIa(R^131^) complex structures than they are to the previously determined FcγRIIa(R^131^) complex structure 3RY6. Our structures may therefore aid in the design of other tailored Fc domains specific for one or more FcγRII receptor.

There are some limitations to our work. We only examined ADCP activity in 4 R^131^ and 5 H^131^ homozygous donors without quantifying FcγRIIa expression levels or the presence of other Fcγ receptors which may confound our comparisons between FcγRIIa alleles. We were also unable to reach saturation in our affinity measurements necessitating kinetic fitting which potentially leads to greater error and reduced confidence in our affinity values. And finally, we were unable to crystallize the Fc(IgG2) with the lower affinity FcγRIIa allele in either species making our conclusions about the hypothetical complexes speculative.

In conclusion, our structural and functional analyses demonstrate that, in humans, the FcγRIIa H^131^ and R^131^ variants exhibit distinct complex architectures with modest differences in IgG subclass affinities, aside from the notable exception of IgG2. In contrast, in macaques, the P^131^ FcγRIIa variant displays uniformly reduced affinity for all IgG subclasses. This potentially provides a basis as to why the H^131^/R^131^ prevalence in humans is roughly equal, while the P^131^ is quite rare. The P^131^ FcγRIIa variant has a much higher threshold for activation which likely translates to lower FcγRIIa mediated immune function and drives the selection for the H^131^ allele; macaques and humans unlike mice only express one low affinity Fcγ receptor on dendritic cells, FcγRIIa (10, 45). Macaques also possess other FcγRIIa polymorphisms absent in humans, underscoring the importance of this and other critical species-specific differences in assessing Fc effector activities in macaques (13–15, 46). These findings have important implications for interpreting FcγRIIa-mediated effector functions in macaque models and for translating such data to human vaccine and antibody infusion studies. In particular, it is important to know that a small subset of macaques, those homozygous for the P^131^ FcγRIIa variant, may have impaired FcγRIIa function which can have an impact in vaccine trials. Likewise, it is important to know that human IgG1 in infusion studies has enhanced FcγRIIa mediated activity relative to macaque IgG1 due to its higher affinity to the macaque FcγRIIa H^131^ allele and to recognize that human and macaque IgG2 are not functionally interchangeable for studies utilizing IgG2.

## Data Availability

The original contributions presented in the study are included in the article/**Supplementary Material**. Further inquiries can be directed to the corresponding author. The datasets and structures generated during the current study are available in the Protein Data Bank (PDB) repository under PDB IDs 9ELW, 9ELZ, 9ELU, 9MCX, 9N5P, 9MCY, and 9OUV.

## References

[1] Schroeder HW Jr. Cavacini L . Structure and function of immunoglobulins. J Allergy Clin Immunol. (2010) 125:S41–52. doi: 10.1016/j.jaci.2009.09.046, PMID: 20176268 PMC3670108

[2] Bruhns P Jonsson F . Mouse and human FcR effector functions. Immunol Rev. (2015) 268:25–51. doi: 10.1111/imr.12350, PMID: 26497511

[3] Chu TH Patz EF Jr. Ackerman ME . Coming together at the hinges: Therapeutic prospects of IgG3. MAbs. (2021) 13:1882028. doi: 10.1080/19420862.2021.1882028, PMID: 33602056 PMC7899677

[4] Vidarsson G Dekkers G Rispens T . IgG subclasses and allotypes: from structure to effector functions. Front Immunol. (2014) 5:520. doi: 10.3389/fimmu.2014.00520, PMID: 25368619 PMC4202688

[5] Lejeune J Brachet G Watier H . Evolutionary story of the low/medium-affinity igG fc receptor gene cluster. Front Immunol. (2019) 10:1297. doi: 10.3389/fimmu.2019.01297, PMID: 31244843 PMC6563257

[6] Tay MZ Wiehe K Pollara J . Antibody-dependent cellular phagocytosis in antiviral immune responses. Front Immunol. (2019) 10:332. doi: 10.3389/fimmu.2019.00332, PMID: 30873178 PMC6404786

[7] Lu LL Suscovich TJ Fortune SM Alter G . Beyond binding: antibody effector functions in infectious diseases. Nat Rev Immunol. (2018) 18:46–61. doi: 10.1038/nri.2017.106, PMID: 29063907 PMC6369690

[8] Neidich SD Fong Y Li SS Geraghty DE Williamson BD Young WC . Antibody Fc effector functions and IgG3 associate with decreased HIV-1 risk. J Clin Invest. (2019) 129:4838–49. doi: 10.1172/JCI126391, PMID: 31589165 PMC6819135

[9] Yuan FF Sullivan JS . FcgammaRIIA polymorphism as a risk factor for invasive Streptococcus pneumoniae. Clin Appl Immunol Rev. (2005) 5:397–403. doi: 10.1016/j.cair.2005.11.001

[10] DiLillo DJ Ravetch JV . Differential fc-receptor engagement drives an anti-tumor vaccinal effect. Cell. (2015) 161:1035–45. doi: 10.1016/j.cell.2015.04.016, PMID: 25976835 PMC4441863

[11] Bruhns P Iannascoli B England P Mancardi DA Fernandez N Jorieux S . Specificity and affinity of human Fcgamma receptors and their polymorphic variants for human IgG subclasses. Blood. (2009) 113:3716–25. doi: 10.1182/blood-2008-09-179754, PMID: 19018092

[12] Chan YN Boesch AW Osei-Owusu NY Emileh A Crowley AR Cocklin SL . IgG binding characteristics of rhesus macaque fcgammaR. J Immunol. (2016) 197:2936–47. doi: 10.4049/jimmunol.1502252, PMID: 27559046 PMC5026948

[13] Conley HE He MM Easterhoff D Kirshner HF Cocklin SL Meyer J . Defining genetic diversity of rhesus macaque Fcgamma receptors with long-read RNA sequencing. Front Immunol. (2023) 14:1306292. doi: 10.3389/fimmu.2023.1306292, PMID: 38264644 PMC10803544

[14] Clatworthy MR . Fcγ Receptor Polymorphisms and Susceptibility to Infection. In: Ackerman ME Nimmerjahn F , editors. Antibody Fc: Linking Adaptive and Innate Immunity. Amsterdam, Elsevier: Academic Press (2014). p. 217–37.

[15] Pollara J Tay MZ Edwards RW Goodman D Crowley AR Edwards RJ . Functional homology for antibody-dependent phagocytosis across humans and rhesus macaques. Front Immunol. (2021) 12:678511. doi: 10.3389/fimmu.2021.678511, PMID: 34093580 PMC8174565

[16] McCoy AJ Grosse-Kunstleve RW Adams PD Winn MD Storoni LC Read RJ . *Phaser* crystallographic software. J Appl Cryst. (2007) 40:658–74. doi: 10.1107/S0021889807021206, PMID: 19461840 PMC2483472

[17] Collaborative Computational Project N . The CCP4 suite: programs for protein crystallography. Acta Crystallogr D Biol Crystallogr. (1994) 50:760–3. doi: 10.1107/S0907444994003112, PMID: 15299374

[18] Emsley P Lohkamp B Scott WG Cowtan K . Features and development of coot. Acta Crystallogr D Biol Crystallogr. (2010) D66:486–501. doi: 10.1107/S0907444910007493, PMID: 20383002 PMC2852313

[19] Adams PD Afonine PV Bunkoczi G Chen VB Davis IW Echols N . PHENIX: a comprehensive Python-based system for macromolecular structure solution. Acta Crystallogr D Biol Crystallogr. (2010) D66:213–21. doi: 10.1107/S0907444909052925, PMID: 20124702 PMC2815670

[20] Chen VB Arendall WB 3rd Headd JJ Keedy DA Immormino RM Kapral GJ . MolProbity: all-atom structure validation for macromolecular crystallography. Acta Crystallogr D Biol Crystallogr. (2010) 66:12–21. doi: 10.1107/S0907444909042073, PMID: 20057044 PMC2803126

[21] Whittle JR Zhang R Khurana S King LR Manischewitz J Golding H . Broadly neutralizing human antibody that recognizes the receptor-binding pocket of influenza virus hemagglutinin. Proc Natl Acad Sci U S A. (2011) 108:14216–21. doi: 10.1073/pnas.1111497108, PMID: 21825125 PMC3161572

[22] Roux KH Strelets L Michaelsen TE . Flexibility of human IgG subclasses. J Immunol. (1997) 159:3372–82. doi: 10.4049/jimmunol.159.7.3372 9317136

[23] Chu TH Crowley AR Backes I Chang C Tay M Broge T . Hinge length contributes to the phagocytic activity of HIV-specific IgG1 and IgG3 antibodies. PloS Pathog. (2020) 16:e1008083. doi: 10.1371/journal.ppat.1008083, PMID: 32092122 PMC7058349

[24] Richardson SI Lambson BE Crowley AR Bashirova A Scheepers C Garrett N . IgG3 enhances neutralization potency and Fc effector function of an HIV V2-specific broadly neutralizing antibody. PloS Pathog. (2019) 15:e1008064. doi: 10.1371/journal.ppat.1008064, PMID: 31841557 PMC6936867

[25] Tolbert WD Gohain N Kremer PG Hederman AP Nguyen DN Van V . Decoding human-macaque interspecies differences in Fc-effector functions: The structural basis for CD16-dependent effector function in Rhesus macaques. Front Immunol. (2022) 13:960411. doi: 10.3389/fimmu.2022.960411, PMID: 36131913 PMC9484259

[26] Caaveiro JM Kiyoshi M Tsumoto K . Structural analysis of Fc/FcgammaR complexes: a blueprint for antibody design. Immunol Rev. (2015) 268:201–21. doi: 10.1111/imr.12365, PMID: 26497522

[27] Crowley AR Richardson SI Tuyishime M Jennewein M Bailey MJ Lee J . Functional consequences of allotypic polymorphisms in human immunoglobulin G subclasses. Immunogenetics. (2023) 75:1–16. doi: 10.1007/s00251-022-01272-7, PMID: 35904629 PMC9845132

[28] Grunst MW Grandea AG 3rd Janaka SK Hammad I Grimes P Karl JA . Functional interactions of common allotypes of rhesus macaque fcgammaR2A and fcgammaR3A with human and macaque igG subclasses. J Immunol. (2020) 205:3319–32. doi: 10.4049/jimmunol.2000501, PMID: 33208458 PMC7725946

[29] Boesch AW Osei-Owusu NY Crowley AR Chu TH Chan YN Weiner JA . Biophysical and functional characterization of rhesus macaque igG subclasses. Front Immunol. (2016) 7:589. doi: 10.3389/fimmu.2016.00589, PMID: 28018355 PMC5153528

[30] Ferrara C Grau S Jager C Sondermann P Brunker P Waldhauer I . Unique carbohydrate-carbohydrate interactions are required for high affinity binding between FcgammaRIII and antibodies lacking core fucose. Proc Natl Acad Sci U S A. (2011) 108:12669–74. doi: 10.1073/pnas.1108455108, PMID: 21768335 PMC3150898

[31] Mizushima T Yagi H Takemoto E Shibata-Koyama M Isoda Y Iida S . Structural basis for improved efficacy of therapeutic antibodies on defucosylation of their Fc glycans. Genes to Cells. (2011) 16:1071–80. doi: 10.1111/j.1365-2443.2011.01552.x, PMID: 22023369 PMC3258418

[32] Patel KR Roberts JT Subedi GP Barb AW . Restricted processing of CD16a/Fc gamma receptor IIIa N-glycans from primary human NK cells impacts structure and function. J Biol Chem. (2018) 293:3477–89. doi: 10.1074/jbc.RA117.001207, PMID: 29330305 PMC5846152

[33] Subedi GP Barb AW . CD16a with oligomannose-type N-glycans is the only "low-affinity" Fc gamma receptor that binds the IgG crystallizable fragment with high affinity *in vitro*. J Biol Chem. (2018) 293:16842–50. doi: 10.1074/jbc.RA118.004998, PMID: 30213862 PMC6204906

[34] Pereira NA Chan KF Lin PC Song Z . The "less-is-more" in therapeutic antibodies: Afucosylated anti-cancer antibodies with enhanced antibody-dependent cellular cytotoxicity. MAbs. (2018) 10:693–711. doi: 10.1080/19420862.2018.1466767, PMID: 29733746 PMC6150623

[35] Dekkers G Treffers L Plomp R Bentlage AEH de Boer M Koeleman CAM . Decoding the human immunoglobulin G-glycan repertoire reveals a spectrum of fc-receptor- and complement-mediated-effector activities. Front Immunol. (2017) 8:877. doi: 10.3389/fimmu.2017.00877, PMID: 28824618 PMC5539844

[36] Crowley AR Osei-Owusu NY Dekkers G Gao W Wuhrer M Magnani DM . Biophysical evaluation of rhesus macaque fc gamma receptors reveals similar igG fc glycoform preferences to human receptors. Front Immunol. (2021) 12:754710. doi: 10.3389/fimmu.2021.754710, PMID: 34712242 PMC8546228

[37] Tolbert WD Subedi GP Gohain N Lewis GK Patel KR Barb AW . From Rhesus macaque to human: structural evolutionary pathways for immunoglobulin G subclasses. MAbs. (2019) 11:709–24. doi: 10.1080/19420862.2019.1589852, PMID: 30939981 PMC6601566

[38] Wang W Maliepaard JCL Damelang T Vidarsson G Heck AJR Reiding KR . Human igG subclasses differ in the structural elements of their N-glycosylation. ACS Cent Sci. (2024) 10:2048–58. doi: 10.1021/acscentsci.4c01157, PMID: 39634222 PMC11613209

[39] Lippold S Mistry K Lenka S Whang K Liu P Pitschi S . Function-structure approach reveals novel insights on the interplay of Immunoglobulin G 1 proteoforms and Fc gamma receptor IIa allotypes. Front Immunol. (2023) 14:1260446. doi: 10.3389/fimmu.2023.1260446, PMID: 37790943 PMC10544997

[40] Ramsland PA Farrugia W Bradford TM Sardjono CT Esparon S Trist HM . Structural basis for Fc gammaRIIa recognition of human IgG and formation of inflammatory signaling complexes. J Immunol. (2011) 187:3208–17. doi: 10.4049/jimmunol.1101467, PMID: 21856937 PMC3282893

[41] Parren PW Warmerdam PA Boeije LC Arts J Westerdaal NA Vlug A . On the interaction of IgG subclasses with the low affinity Fc gamma RIIa (CD32) on human monocytes, neutrophils, and platelets. Analysis of a functional polymorphism to human IgG2. J Clin Invest. (1992) 90:1537–46. doi: 10.1172/JCI116022, PMID: 1401085 PMC443201

[42] Dillon TM Ricci MS Vezina C Flynn GC Liu YD Rehder DS . Structural and functional characterization of disulfide isoforms of the human IgG2 subclass. J Biol Chem. (2008) 283:16206–15. doi: 10.1074/jbc.M709988200, PMID: 18339626 PMC3259628

[43] Zhang B Harder AG Connelly HM Maheu LL Cockrill SL . Determination of Fab-hinge disulfide connectivity in structural isoforms of a recombinant human immunoglobulin G2 antibody. Anal Chem. (2010) 82:1090–9. doi: 10.1021/ac902466z, PMID: 20039682

[44] Mimoto F Katada H Kadono S Igawa T Kuramochi T Muraoka M . Engineered antibody Fc variant with selectively enhanced FcgammaRIIb binding over both FcgammaRIIa(R131) and FcgammaRIIa(H131). Protein Eng Des Sel. (2013) 26:589–98. doi: 10.1093/protein/gzt022, PMID: 23744091 PMC3785249

[45] O'Doherty U Ignatius R Bhardwaj N Pope M . Generation of monocyte-derived dendritic cells from precursors in rhesus macaque blood. J Immunol Methods. (1997) 207:185–94. doi: 10.1016/S0022-1759(97)00119-1, PMID: 9368645

[46] Tuyishime M Spreng RL Hueber B Nohara J Goodman D Chan C . Multivariate analysis of FcR-mediated NK cell functions identifies unique clustering among humans and rhesus macaques. Front Immunol. (2023) 14:1260377. doi: 10.3389/fimmu.2023.1260377, PMID: 38124734 PMC10732150

