# Supplementary material for: Cross-species analysis of FcγRIIa/b (CD32a/b) polymorphisms at position 131: structural and functional insights into the mechanism of IgG- mediated phagocytosis in human and macaque
Source: Front Immunol. 2025 Dec 8;16:1726068. doi: 10.3389/fimmu.2025.1726068 (PMC12723028; doi:10.3389/fimmu.2025.1726068)
Supplement: Supplementary file 1 [file DataSheet1.pdf]

Supplementary Material

1 Supplementary Figures and Tables

1.1 Supplementary Tables

Supplementary Table S1: Mean K_D values and standard deviations for human and macaque VRC01 IgG1, 2, 3, and 4 against human and macaque FcγRIIa variants, H^{131} and R^{131} for human and H^{131} and P^{131} for macaque, and human FcγRIIb. The number of technical replicates for each reported value is in parentheses.

	Hs **FcγRIIa(H^{131})**	***Hs*** **FcγRIIa(R^{131})**	***Hs*** **FcγRIIb(R^{131})**	***Mm*** **FcγRIIa(H^{131})**	***Mm*** **FcγRIIa(P^{131})**
Hs **IgG1**	6.4 ± 1.1 µM (3)	11.4 ± 4.9 µM (4)	11.1 ± 0.8 µM (4)	5.3 ± 0.8 µM (4)	33.4 ± 15.8 µM (4)
Hs **IgG2**	11.2 ± 3.2 µM (4)	10.4 ± 3.2 µM (3)	29.9 ± 4.8 µM (4)	13.8 ± 2.8 µM (3)	99 ± 17 µM (3)
Hs **IgG3**	12.5 ± 0.6 µM (4)	14.2 ± 4.2 µM (4)	7.5 ± 1.1 µM (4)	11 ± 4.7 µM (4)	82.8 ± 23 µM (4)
Hs **IgG4**	43.6 ± 8.8 µM (4)	15.2 ± 1.8 µM (4)	8.7 ± 1.3 µM (4)	18.2 ± 1.8 µM (4)	41.8 ± 14.3 µM (4)
Mm **IgG1**	9.6 ± 2.5 µM (4)	11 ± 2.4 µM (4)	–	7.8 ± 1.2 µM (4)	44.7 ± 8.4 µM (3)
Mm **IgG2**	9.4 ± 0.5 µM (4)	22.1 ± 6.6 µM (3)	–	9.1 ± 0.7 µM (4)	26.3 ± 13 µM (3)
Mm **IgG3**	16.9 ± 0.8 µM (3)	8.1 ± 0.8 µM (4)	–	13.3 ± 5.8 µM (3)	34 ± 10 µM (4)
Mm **IgG4**	13.6 ± 4.0 µM (4)	7.3 ± 0.9 µM (4)	–	21 ± 5.0 µM (3)	102 ± 28 µM (4)

Supplementary Table S2. Data collection and refinement statistics.

	Mm FcγRIIa(H[131])-IgG1Fc complex	*Mm* FcγRIIa(H[131])-IgG2Fc complex	*Mm* FcγRIIa(P[131])-IgG1Fc complex	*Hs* FcγRIIa(H[131])-IgG1Fc complex	*Hs* FcγRIIa(H[131])-IgG2Fc complex	*Hs* FcγRIIa(R[131])-IgG1Fc complex	*Hs* FcγRIIb-IgG1Fc complex
**Data collection**							
Wavelength, (Å)	0.979	0.979	0.979	0.979	0.979	0.979	0.979
Space group	$P6_522$	$P6_522$	$P6_522$	C2	C2	P1	$P2_1$
Cell parameters							
a, b, c, Å	127.2, 127.2, 254.2	128.1, 128.1, 253.5	127.5, 127.5, 256.1	161.1, 66.1, 72.1	161.9, 66.9, 71.2	49.0, 76.2, 120.0	49.4, 76.1, 120.7
α, β, γ, °	90, 90, 120	90, 90, 120	90, 90, 120	90, 94.3, 90	90, 94.2, 90	96.6, 100.2, 90.1	90, 95.5, 90
Complexes/a.u.	1	1	1	1	1	2	1
Resolution, (Å)	50-3.55 (3.61-3.55)	50-3.2 (3.26-3.2)	50-2.65 (2.7-2.65)	50-2.38 (2.42-2.38)	50-2.0 (2.03-2.0)	50-2.85 (2.9-2.85)	50-3.07 (3.12-3.07)
# of reflections							
Total	262,194	380,536	671,846	99,631	161,374	122,829	55,338
Unique	15,333	21,259	36,316	28,466	44,826	38,384	16,276
R_{merge}[a], %	34.8 (100)	21.2 (100)	12.3 (100)	6.8 (21.9)	13.5 (49.3)	17.9 (59.6)	15.4 (79.4)
R_{pim}[b], %	8.6 (91.8)	4.8 (48.1)	2.9 (64.7)	4.2 (13.9)	7.8 (33.9)	11.2 (47.8)	9.4 (57.4)
$CC_{1/2}$[c]	0.98 (0.52)	0.99 (0.48)	1.0 (0.36)	0.99 (0.95)	0.99 (0.63)	0.76 (0.64)	0.95 (0.48)
I/σ	28.5 (0.9)	17.9 (0.7)	57.5 (0.9)	43.9 (5.9)	28.7 (1.8)	12.5 (1.5)	9.9 (1.3)
Completeness, %	99.9 (99.2)	98.2 (77.0)	99.9 (98.2)	93.5 (93.5)	89.5 (77.2)	95.6 (70.1)	95.9 (84.5)
Redundancy	17.1 (14.1)	17.8 (5.4)	18.5 (12.0)	3.5 (3.2)	3.6 (2.6)	3.2 (2.1)	3.4 (2.3)
**Refinement Statistics**							
Resolution, (Å)	50.0 – 3.55	50.0 – 3.2	50.0 – 2.65	50.0 – 2.38	50.0 – 2.0	50.0 – 2.85	50.0 – 3.07
R[d] %	29.3	27.5	27.7	18.5	19.5	17.7	18.6
R_{free}[e], %	33.7	30.1	32.7	23.4	23.2	23.6	24.1
# of atoms							
Protein	4,769	4,767	4,795	4,742	4,738	9,538	4,790
Water	–	–	2	216	281	11	1
Ligand/glycan	282	268	261	261	261	468	254
Overall B value $(Å)^2$							
Protein	196	145	153	38	51	72	71
Water	–	–	79	37	48	44	23
Ligand/glycan	209	149	174	56	69	85	90
RMSD[f]							
Bond lengths, Å	0.013	0.009	0.011	0.005	0.006	0.010	0.004
Bond angles, °	1.75	1.3	1.45	0.8	0.85	1.2	0.8
Ramachandran[g]							
favored, %	70.1	70.1	74.5	94.7	96.6	91.4	89.8
allowed, %	18.5	19.7	17.1	3.6	2.2	7.1	7.9
outliers, %	11.4	10.3	8.4	1.7	1.2	1.5	2.3
PDB ID	9ELW	9ELZ	9ELU	9MCX	9N5P	9MCY	9OUV

Values in parentheses are for highest-resolution shell

[a]$R_{merge} = \sum|I - \langle I\rangle|/\sum I$, where I is the observed intensity and $\langle I\rangle$ is the average intensity obtained from multiple observations of symmetry-related reflections after rejections

[b]R_{pim} = as defined in (1)

[c]$CC_{1/2}$ = as defined by Karplus and Diederichs (2)

[d]$R = \sum||F_o| - |F_c||/\sum|F_o|$, where F_o and F_c are the observed and calculated structure factors, respectively

[e]R_{free} = as defined by Brünger (3)

[f]RMSD = Root mean square deviation

[g]Calculated with MolProbity

Supplementary Table S3: Details of the human FcγRIIa(H^{131})-IgG1Fc, FcγRIIa(H^{131})-IgG2Fc, and FcγRIIa(R^{131})-IgG1Fc complexes and the macaque FcγRIIa(H^{131})-IgG1Fc, FcγRIIa(H^{131})-IgG2Fc, and FcγRIIa(P^{131})-IgG1Fc complexes. Buried surface areas (BSAs) were calculated using the EBI PISA server (http://www.ebi.ac.uk/msd-srv/prot_int/cgi-bin/piserver). BSA contributions from the Fc are split into contributions from chain A and chain B of the Fc dimer. The human FcγRIIa(R^{131})-IgG1Fc BSA contributions are an average of the two complexes in the asymmetric unit. Values in parenthesis represent the contributions from glycans.

		Hs FcγRIIa(H^{131}) -IgG1Fc (PDB 9MCX)		*Hs* FcγRIIa(H^{131}) -IgG2Fc (PDB 9N5P)		*Hs* FcγRIIa(R^{131}) - IgG1Fc (PDB 9MCY)		*Mm* FcγRIIa(H^{131}) -IgG1Fc (PDB 9ELW)		*Mm* FcγRIIa(H^{131}) - IgG2Fc (PDB 9ELZ)		*Mm* FcγRIIa(P^{131}) - IgG1Fc (PDB 9ELU)	
**Buried Surface Area, Å^2**		**A**	**B**	**A**	**B**	**A**	**B**	**A**	**B**	**A**	**B**	**A**	**B**
	**Fc total**	**504 (30)**	**392 (0)**	**535 (30)**	**415 (0)**	**610 (75)**	**388 (5)**	**491 (51)**	**311 (0)**	**498 (48)**	**333 (0)**	**519 (70)**	**319 (0)**
	Fc protein	474	392	505	415	535	383	440	311	450	333	449	319
	Fc lower hinge region	175	196	215	223	202	181	195	93	210	92	240	85
	Fc BC loop	91	0	88	0	106	0	71	10	56	7	51	3
	Fc DE loop	177	0	178	0	232	5	166	0	165	0	184	0
	Fc FG loop	19	195	11	193	24	202	17	208	22	210	11	231
	Fc glycan(Asn^{297})	30	0	30	0	75	5	51	0	48	0	70	0
	**FcγRIIa total**	**523 (25)**	**329 (0)**	**502 (25)**	**341 (0)**	**599 (62)**	**320 (5)**	**507 (42)**	**261 (0)**	**513 (40)**	**278 (0)**	**546 (59)**	**257 (0)**
	FcγRIIa protein	498	329	477	341	537	315	465	261	473	278	487	257
	**FcγRIIa and Fc total**	**1027 (55)**	**721 (0)**	**1037 (55)**	**756 (0)**	**1209 (137)**	**708 (10)**	**998 (93)**	**572 (0)**	**1011 (88)**	**611 (0)**	**1065 (129)**	**576 (0)**
		**1748 (55)**		**1793 (55)**		**1917 (147)**		**1570 (93)**		**1622 (88)**		**1641 (129)**	

Supplementary Table S4: Details of the human FcγRIIa(R^{131})-IgG1Fc and FcγRIIb-IgG1Fc from this paper, the FcγRIIa(R^{131})-IgG1Fc complex from PDB ID 3RY6, the FcγRIIb-IgG1(P^{238}D)Fc from 3WJJ, and the FcγRIIb-IgG1(V12)Fc from 3WJL. The version 12 (V12) IgG1Fc from 3WJL has five additional mutations in addition to P^{238}D to enhance FcγRIIb binding: E^{233}D, G^{237}D, H^{268}D, P^{271}G and A^{330}R (4). Buried surface areas were calculated using the EBI PISA server (http://www.ebi.ac.uk/msd-srv/prot_int/cgi-bin/piserver). BSA contributions from the Fc are split into contributions from chain A and chain B of the Fc dimer. The human FcγRIIa(R^{131})-IgG1Fc BSA contributions from PDB ID 9MCY are an average of the two complexes in the asymmetric unit. Values in parenthesis represent the contributions from glycans.

		Hs FcγRIIa(R^{131}) - IgG1Fc (PDB 9MCY)		*Hs* FcγRIIa(R^{131}) - IgG1Fc (PDB 3RY6)		*Hs* FcγRIIb - IgG1Fc (PDB 9OUV)		*Hs* FcγRIIb - IgG1(P^{238D})Fc (PDB 3WJJ)		*Hs* FcγRIIb - IgG1(V12)Fc (PDB 3WJL)	
**Buried Surface Area, Å²**		**A**	**B**	**A**	**B**	**A**	**B**	**A**	**B**	**A**	**B**
	**Fc total**	**610 (75)**	**388 (5)**	**526 (165)**	**310 (0)**	**620 (59)**	**411 (0)**	**339 (55)**	**329 (0)**	**362 (71)**	**395 (0)**
	Fc protein	535	383	361	310	561	411	284	329	291	395
	Fc lower hinge region	202	181	142	153	229	208	0	118	0	174
	Fc BC loop	106	0	25	0	107	0	92	0	72	0
	Fc DE loop	232	5	260	0	215	0	180	0	213	0
	Fc FG loop	24	202	0	158	21	229	34	210	27	220
	Fc glycan(Asn^{297})	75	5	165	0	59	0	55	0	71	0
	**FcγRIIa/b total**	**599 (62)**	**320 (5)**	**565 (139)**	**284 (0)**	**645 (50)**	**318 (0)**	**358 (40)**	**311 (0)**	**366 (56)**	**390 (0)**
	FcγRIIa/b protein	537	315	426	284	595	318	318	311	310	390
	**FcγRIIa/b and Fc total**	**1209 (137)**	**708 (10)**	**1091 (304)**	**594 (0)**	**1265 (109)**	**729 (0)**	**697 (95)**	**640 (0)**	**728 (127)**	**785 (0)**
		**1917 (147)**		**1685 (304)**		**1994 (109)**		**1337 (95)**		**1513 (127)**	

1.2 Supplementary Figures

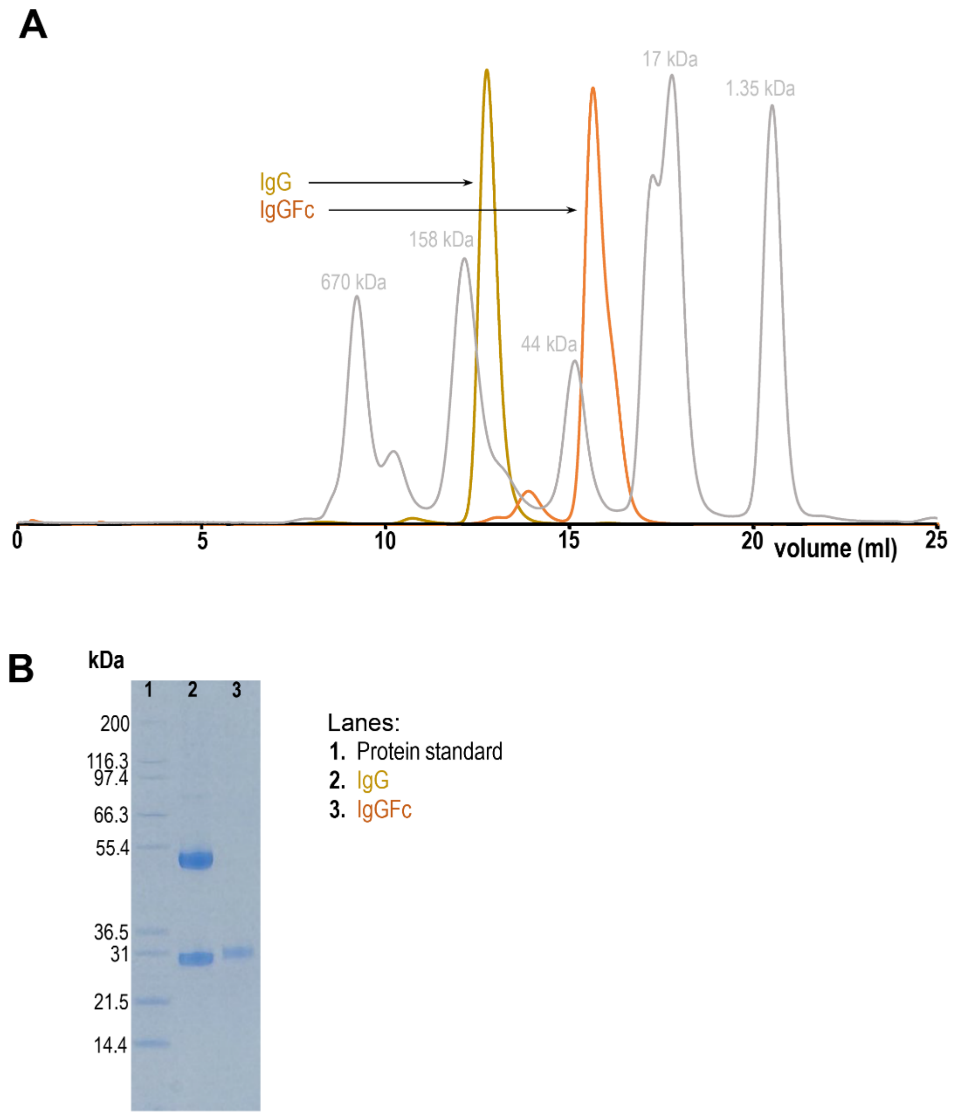

Supplementary Figure S1. (**A**) Superposition of chromatographs for IgG1 (yellow) and IgG1Fc (orange). Chromatographs of IgG2 and IgG2Fc look similar. BioRad chromatograph standards are shown in grey with molecular weights as indicated. (**B**) SDS-PAGE gel of peaks from size exclusion chromatography labeled as shown. Mark12 SDS-PAGE standards are shown for reference in lane 1 with sizes as indicated.

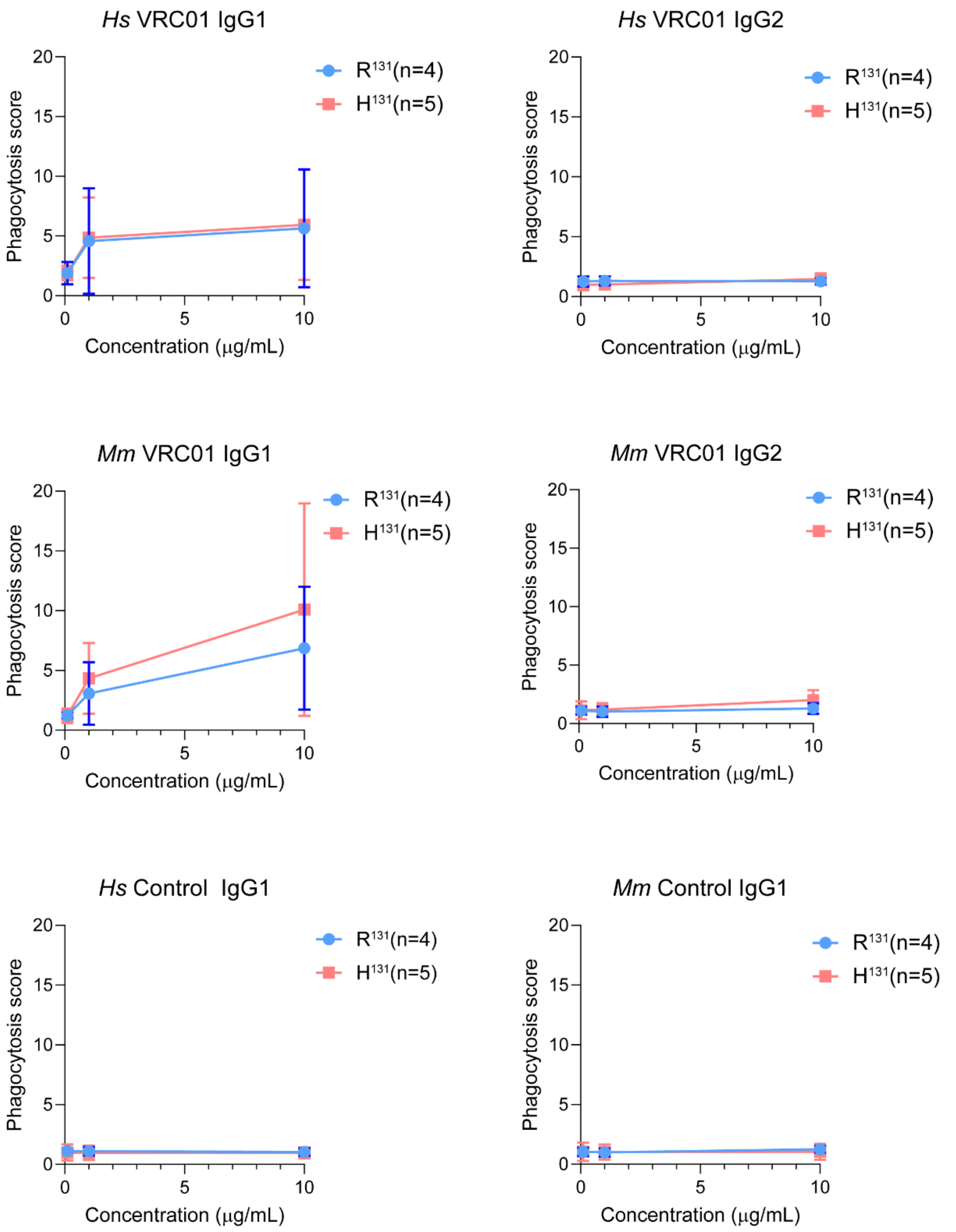

Supplementary Figure S2. **ADCP activity as a function of Ab concentration.** ADCP activity measured as a phagocytosis score for human and macaque VRC01 IgG1 and IgG2 using human monocytes homozygous for the R^{131} or H^{131} FcγRIIa allele. The human influenza CH65 IgG1 is used as a control. Measurements are shown as the mean plus or minus the standard deviation.

Alt. text: Multi-panel figure showing ADCP activity as a function of Ab concentration for VRC01 IgG1 and IgG2 as compared to the human influenza CH65 IgG1 control.

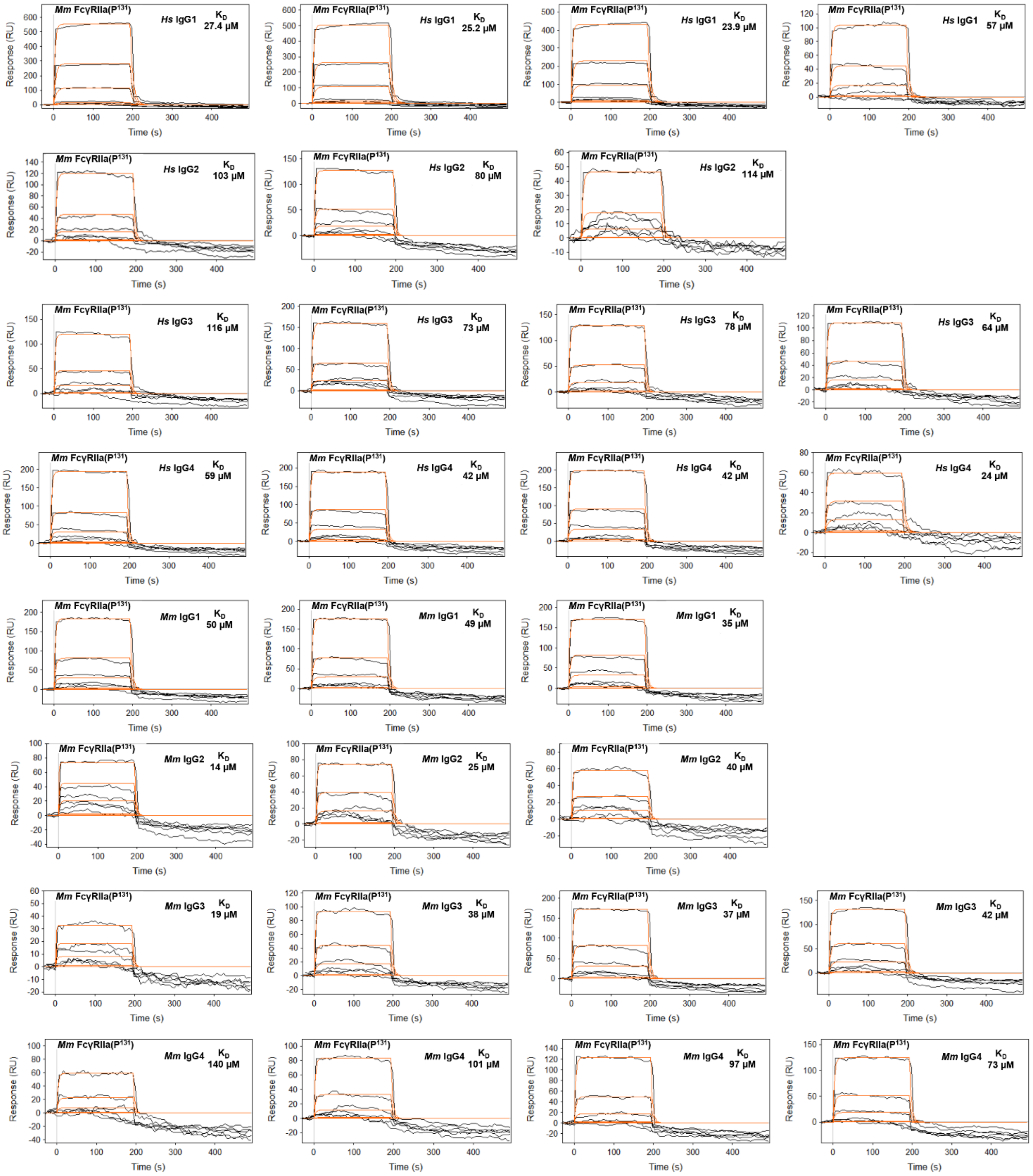

Supplementary Figure S3. SPR sensorgrams for macaque FcγRIIa(P^{131}) to human and macaque IgG1, 2, 3, and 4. The reference subtracted sample response is in black and the best fit to 1:1 binding stoichiometry is in orange. Each curve series represents one independent K_D determination.

Alt. text: Multi-panel figure showing the binding curves of macaque FcγRIIa(P^{131}) to human and macaque IgG1, 2, 3, and 4. Each curve series represents one independent K_D determination.

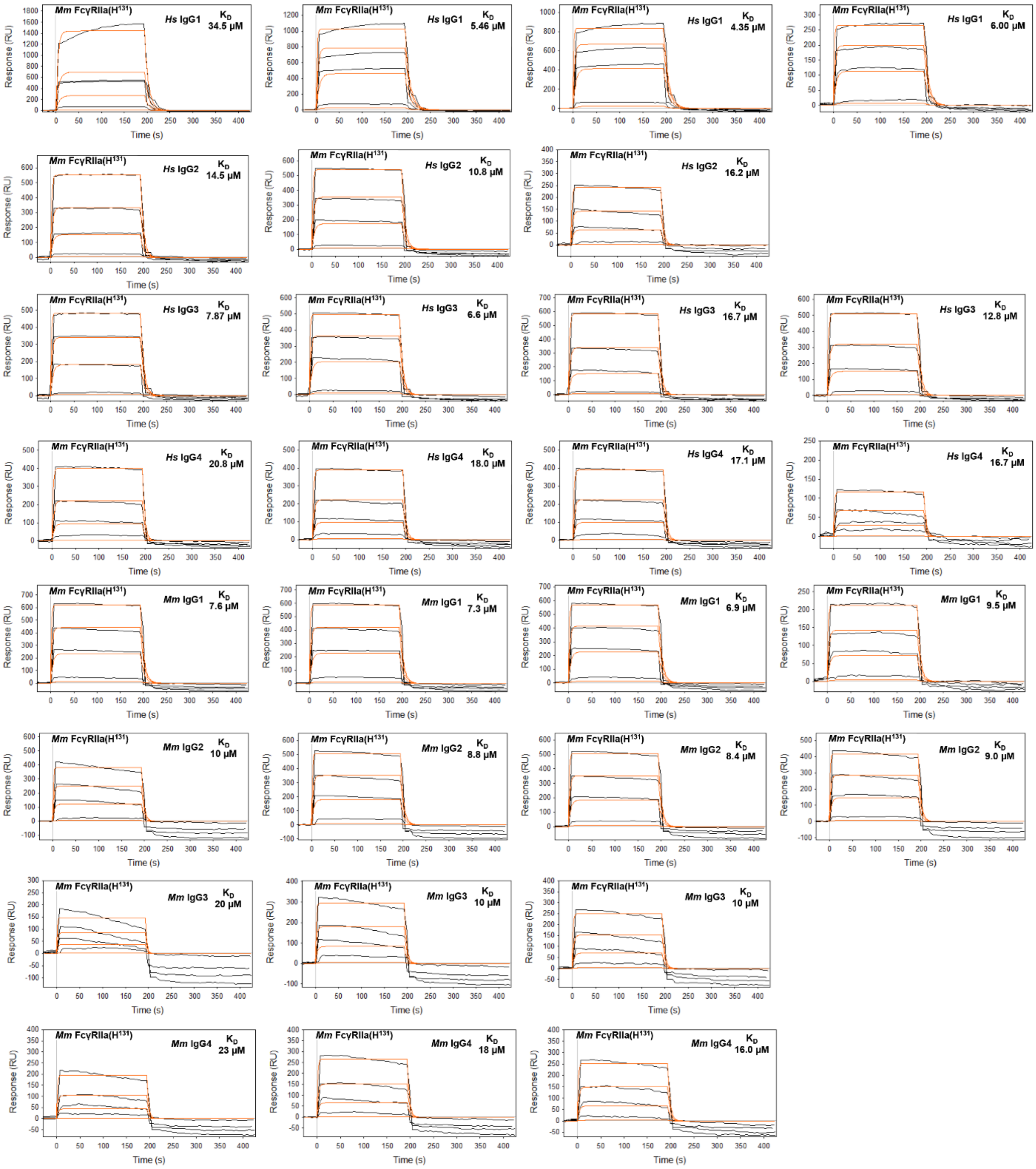

Supplementary Figure S4. SPR sensorgrams for macaque FcγRIIa(H^{131}) to human and macaque IgG1, 2, 3, and 4. The reference subtracted sample response is in black and the best fit to 1:1 binding stoichiometry is in orange. Each curve series represents one independent K_D determination.

Alt. text: Multi-panel figure showing the binding curves of macaque FcγRIIa(H^{131}) to human and macaque IgG1, 2, 3, and 4. Each curve series represents one independent K_D determination.

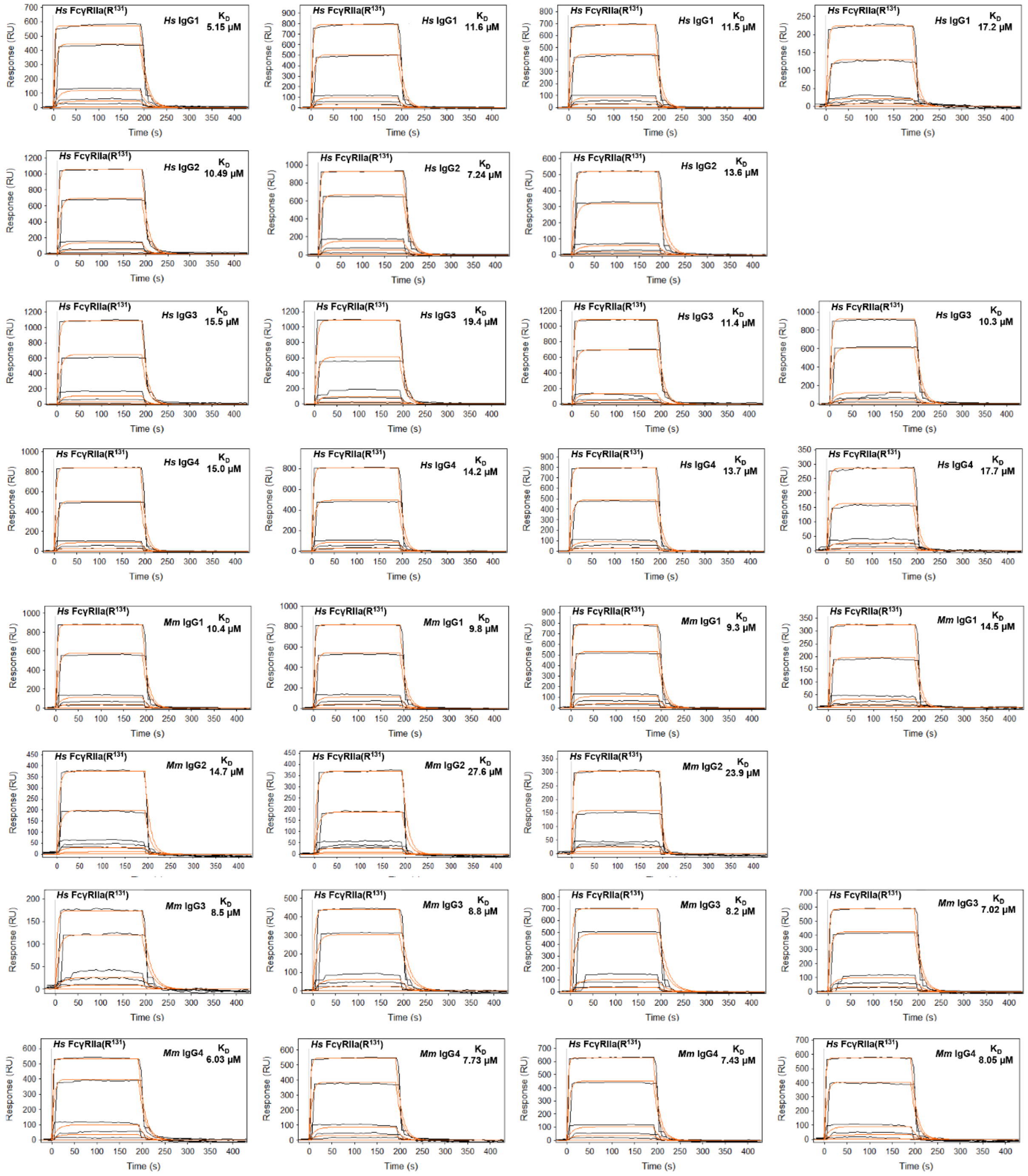

Supplementary Figure S5. SPR sensorgrams for human FcγRIIa(R^{131}) to human and macaque IgG1, 2, 3, and 4. The reference subtracted sample response is in black and the best fit to 1:1 binding stoichiometry is in orange. Each curve series represents one independent K_D determination.

Alt. text: Multi-panel figure showing the binding curves of human FcγRIIa(R^{131}) to human and macaque IgG1, 2, 3, and 4. Each curve series represents one independent K_D determination.

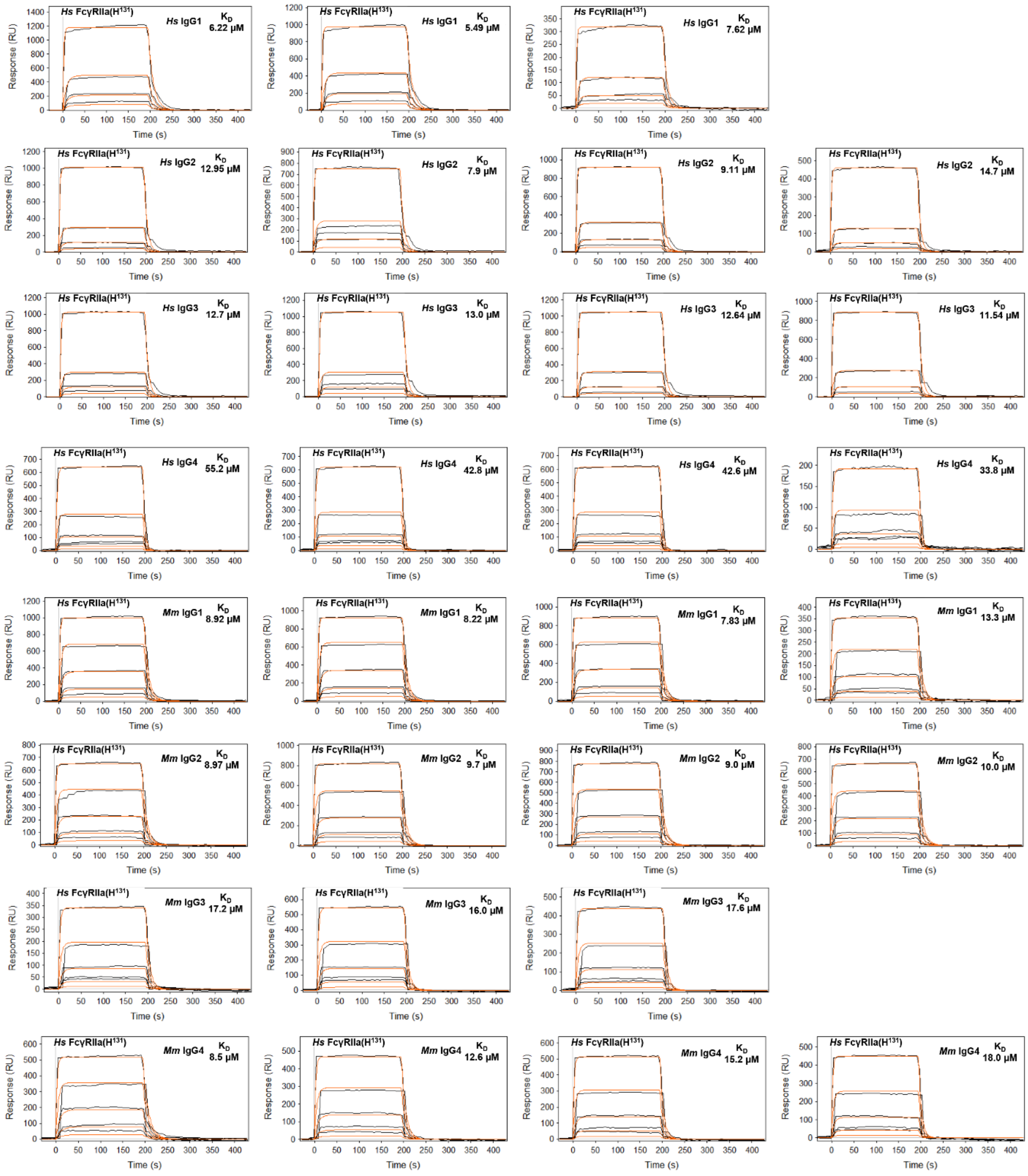

Supplementary Figure S6. SPR sensorgrams for human FcγRIIa(H^{131}) to human and macaque IgG1, 2, 3, and 4. The reference subtracted sample response is in black and the best fit to 1:1 binding stoichiometry is in orange. Each curve series represents one independent K_D determination.

Alt. text: Multi-panel figure showing the binding curves of human FcγRIIa(H^{131}) to human and macaque IgG1, 2, 3, and 4. Each curve series represents one independent K_D determination.

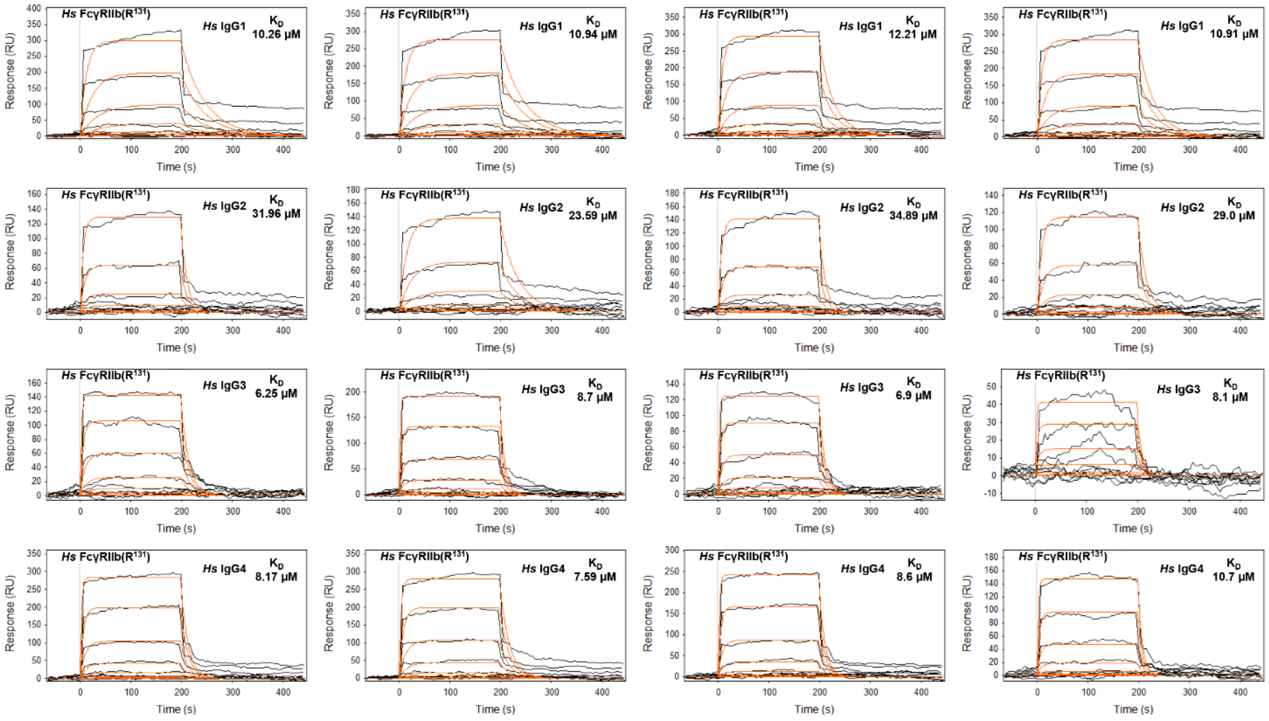

Supplementary Figure S7. SPR sensorgrams for human FcγRIIb(R^{131}) to human IgG1, 2, 3, and 4. The reference subtracted sample response is in black and the best fit to 1:1 binding stoichiometry is in orange. Each curve series represents one independent K_D determination.

Alt. text: Multi-panel figure showing the binding curves of human FcγRIIb(R^{131}) to human IgG1, 2, 3, and 4. Each curve series represents one independent K_D determination.

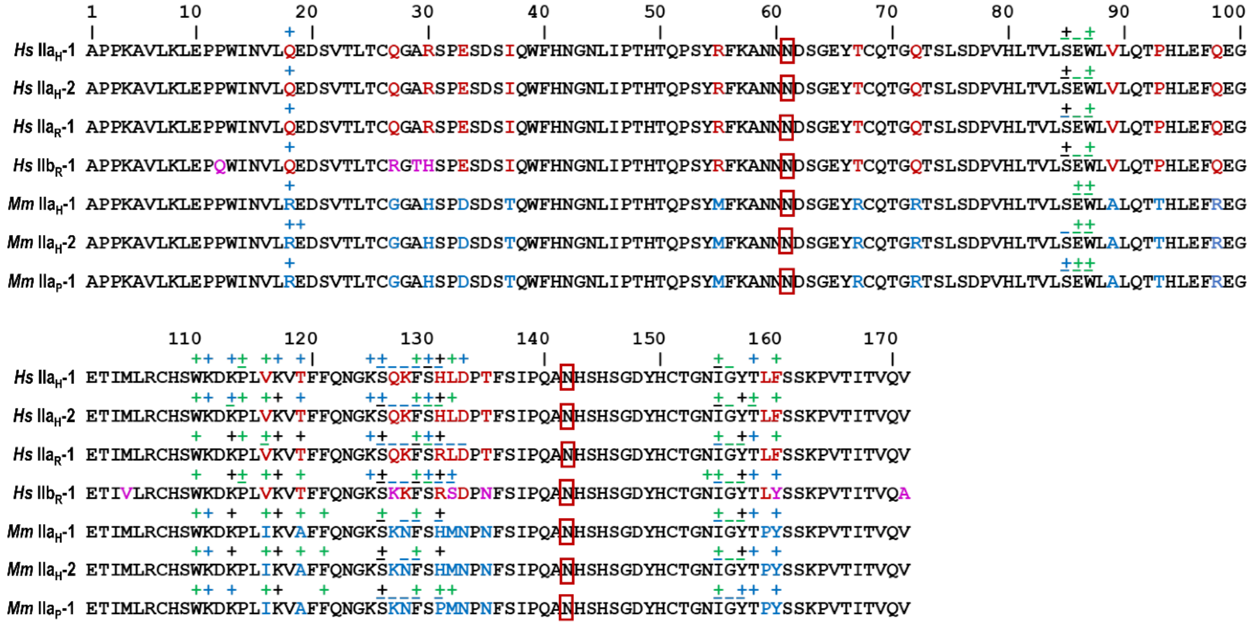

Supplementary Figure S8. Sequence alignment of FcγRIIa/b receptors. Receptor contact residues defined by a 5 Å cutoff are marked above the sequence with (+) for side chain and (-) for main chain to indicate the type of contact. Contact types are colored as follows: hydrophilic (blue), hydrophobic (green) and both (black). Sequence differences in human versus macaque are shown in red and blue respectively, and sequence differences in human FcγRIIb as compared to human FcγRIIa are shown in violet. Asparagines with N-linked glycans are marked with a red box.

Alt. text: Sequence alignment of the human and macaque FcγRIIa/b receptors. Identical residues across all receptors are shown in black, while those that differ between human and macaque or FcγRIIa and FcγRIIb are shown in red or blue, respectively. Asparagines with N-linked glycans are marked with a red box.

1.3 Supplementary References

1. Weiss MS. Global indicators of X-ray data quality. Applied Crystallography. 2001;34(2):130-5.

2. Karplus PA, Diederichs K. Linking crystallographic model and data quality. Science. 2012;336(6084):1030-3.

3. Brunger AT. Free *R* value: Cross-validation in crystallography. Methods in Enzymology. 277: Academic Press; 1997. p. 366-96.

4. Adams PD, Afonine P, Bunkoczi G, Chen V, Davis I, Echols N. PHENIX: a comprehensive Python-based system for macromolecular structure solution Acta Crystallogr D Biol Crystallogr 66. 2010.
